# Adherence to the Planetary Health Diet Index and Fetal Body Composition

**DOI:** 10.1001/jamanetworkopen.2025.44153

**Published:** 2025-12-17

**Authors:** Priscilla K. Clayton, Elizabeth A. DeVilbiss, Shan-Xuan Lim, Jessica L. Gleason, Zhen Chen, Dian He, Roger B. Newman, Katherine L. Grantz, Edwina H. Yeung, Jagteshwar Grewal

**Affiliations:** 1 Epidemiology Branch, Division of Population Health Research, Eunice Kennedy Shriver National Institute of Child Health and Human Development, National Institutes of Health, Bethesda, Maryland; 2 Division of Population Health Research, Division of Intramural Research, Eunice Kennedy Shriver National Institute of Child Health and Human Development, National Institutes of Health, Bethesda, Maryland; 3 Biostatistics and Bioinformatics Branch, Division of Population Health Research, Division of Intramural Research, Eunice Kennedy Shriver National Institute of Child Health and Human Development, National Institutes of Health, Bethesda, Maryland; 4 The Prospective Group Inc, Arlington, Virginia; 5 Department of Obstetrics and Gynecology, Medical University of South Carolina, Charleston

## Abstract

**Importance:**

The Planetary Health Diet (PHD), introduced by the EAT-*Lancet* Commission in 2019, emphasizes a plant-based diet. Several cohorts have assessed adherence using the PHD Index (PHDI), but evidence is limited on whether maternal periconceptional and early pregnancy adherence is associated with fetal growth.

**Objective:**

To examine the association of maternal PHDI adherence in early pregnancy with longitudinal 2-dimensional and 3-dimensional fetal biometric measures.

**Design, Setting, and Participants:**

Secondary analysis of a prospective cohort of pregnant women with singletons (July 2009 to January 2013) at 12 US clinical sites. Women with preexisting health conditions were excluded. Participants had data on fetal body composition and organ volumes (April 2016 to September 2019). Analyses were conducted June 2024 to August 2025.

**Exposure:**

PHDI adherence categorized as low, moderate, or high, assessed by the Food Frequency Questionnaire between 8 to 13 weeks’ gestation.

**Main Outcomes and Measures:**

Estimated fetal weight (EFW), head circumference (HC), humerus and femur lengths, abdominal circumference and area, abdominal subcutaneous tissue thickness, fractional thigh and arm volumes (total, lean, and fat), midthigh areas, ratio of fractional fat thigh volume to fractional thigh volume, and ratio of midthigh fat area to midthigh area, measured up to 5 times between 15 to 42 weeks’ gestation. Trajectories were modeled using linear mixed-effects models.

**Results:**

Analyses included 1464 women. Mean (SD) maternal age was 28.1 (5.6) years, and mean (SD) gestational age at delivery was 39.2 (1.7) weeks. High vs low PHDI adherence was associated with larger EFW at 32 to 40 weeks (32 weeks: difference, 35 g; 95% CI, 22 to 49 g; 40 weeks: difference, 165 g; 95% CI, 108 to 223 g), larger HC at 37 to 39 weeks (37 weeks: difference, 1.89 mm; 95% CI, 1.10 to 2.69 mm; 39 weeks: difference, 2.44 mm; 95% CI, 1.47 to 3.42 mm), smaller fractional lean arm volume at 34 to 37 weeks; (34 weeks: difference, −0.40 cm^3^; 95% CI, −0.56 to −0.25 cm^3^), larger fractional fat arm volume at 28 to 29 weeks; (28 weeks: difference, 0.23 cm^3^; 95% CI, 0.13 to 0.34 cm^3^), and larger abdominal area at 25 to 26 weeks (25 weeks: difference, 62.2 mm^2^; 95% CI, 34.4 to 90.0 cm^3^). Moderate adherence showed similar patterns.

**Conclusions and Relevance:**

In this cohort study of pregnant women, higher maternal PHDI adherence in early pregnancy was associated with greater fetal adiposity and reduced lean tissue, suggesting potential implications for offspring metabolic health.

## Key Points

**Question:**

Is periconceptional and early pregnancy adherence to the Planetary Health Diet (PHD) associated with fetal growth?

**Findings:**

In this cohort study of 1464 pregnant women, high relative to low maternal adherence to the PHD was associated with larger fetal fractional fat arm volumes in the second trimester and larger fetal weight and abdominal measures in the third trimester.

**Meaning:**

In this cohort study, adherence to the PHD during periconceptional and early pregnancy was associated with larger in utero fetal adiposity measures in the second and third trimester, suggesting potential implications for offspring metabolic health.

## Teaser

This cohort study assesses associations of low, moderate, and high adherence to the Planetary Health Diet Index among pregnant women with measures of fetal body composition and organ volumes.

## Introduction

In the US, approximately 1 in 5 children had obesity between 2017 and 2020, affecting more than 47 million children.^1^ The Developmental Origins of Health and Disease framework emphasizes that exposures during critical periods of fetal development, including maternal nutrition, are associated with offspring growth and long-term health outcomes, with evidence from US-based studies supporting these associations.^2,3^ Poor maternal nutritional status during pregnancy has received considerable attention in previous studies examining fetal growth, as well as being associated with childhood obesity for the offspring.^4,5^ In 2019, the EAT-*Lancet* Commission developed a Planetary Health Diet (PHD), a plant-based diet with moderate amounts of animal-based foods.^6^ Since then, several cohort studies have quantified the PHD by deriving the PHD Index (PHDI). The PHD recommendations are based on the environmental impact of each food group. Meanwhile, no evidence is currently available about whether adhering to this diet influences fetal development.

Several studies have investigated associations of other maternal dietary patterns with neonatal anthropometry. One study^7^ found vegetarian diets during pregnancy were associated with smaller neonates, specifically with lower mean birthweight and length, when compared with nonvegetarian diets. Another study^8^ found greater adherence (quartile 4 vs quartile 1) to the Alternative Healthy Eating Index-2010, the Alternate Mediterranean Diet Score, and the Dietary Approaches to Stop Hypertension, associated with a mean 97- to 100-g increase in birth weight.

Relevant research typically examines birth size, whereas assessment focusing on longitudinal pattern of in utero fetal body composition is less common.^9,10^ Select studies have examined the association of in utero fetal body composition with maternal adherence to dietary patterns. For instance, Rovira et al^5^ found no association of adherence to the Mediterranean diet among 542 pregnant women with 2-dimensional (2D) ultrasonography measures. Quin et al^4^ found adherence to dietary patterns rich in vegetables and fish was associated with lower fetal head circumference (HC) among 1936 pregnant women.

Whether adherence to the PHDI influences fetal growth remains unclear. We aimed to examine associations of the PHDI and fetal body composition across pregnancy using 2D and 3-dimensional (3D) measurements. Longitudinal evaluation of fetal growth trajectories by serial ultrasonography may better reflect metabolic programming and help identify when differences in growth occur.^11^ Furthermore, 3D ultrasonography technology allows for measurement of fetal organ and limb volumes, as well as muscle, bone, and fat, in ways not possible with 2D ultrasonography.^12^ Maternal adherence to the PHDI may be associated with differences in 3D organ and limb volume growth that are not apparent using 2D fetal measures.

## Methods

### Study Population

Institutional review board approval was obtained for this cohort study by all participating institutions, the data coordinating center, and the Eunice Kennedy Shriver National Institute of Child Health and Human Development (NICHD). This work followed the Strengthening the Reporting of Observational Studies in Epidemiology (STROBE) reporting guideline. The NICHD Fetal 3D Study (2015-2019) comprised women originally recruited in the NICHD Fetal Growth Studies, a racially diverse prospective cohort that enrolled pregnant women at 12 US clinical sites (July 2009 to January 2013). The primary aim of the prospective cohort was to establish fetal growth standards for 4 self-identified racial and ethnic groups (Asian or Pacific Islander, Hispanic, non-Hispanic Black, and non-Hispanic White).^13^ Longitudinal US data were collected from 2334 women with relatively low-risk pregnancies who fell within the normal or overweight range (prepregnancy body mass index [BMI; calculated as weight in kilograms divided by height in meters squared]: 19.0-29.9) and another 468 women with obesity (prepregnancy BMI: 30.0-44.9)—all without major preexisting health conditions. Mothers were considered eligible for the study if they had a singleton pregnancy, were aged 18 to 40 years, with a known last menstrual period date, without confirmed or suspected fetal congenital structural or chromosomal anomalies, and expected to deliver at a participating hospital. Additional details about the recruitment, the inclusion and exclusion criteria, and the data collection have been previously reported.^14^

Mothers were enrolled at 8 to 13 weeks’ gestation and followed up through delivery, with ultrasonography measurements taken at up to 5 study visits.^13^ Of the 2802 enrolled women, a subset of 1980 women were invited to participate in the nutrition component of the study, which was deployed midway through recruitment.^15^ Written consent was obtained from all participants before data collection.

### Measurements

#### Dietary Assessments

At enrollment between 8 to 13 weeks’ gestation, mothers were asked to complete a self-administered, semiquantitative, 139-item Food Frequency Questionnaire (FFQ) to assess maternal diet during the past 3 months. The FFQ was derived from the modified version of the Diet History Questionnaire-II.^16^ Mothers were asked to report how often they usually consumed each food, expressed in terms of the number of times per day, week, or month, and the usual portion size. Nutrient intakes and My Pyramid Equivalent Database for each respective FFQ item were obtained using the Diet*Calc Software version 1.5.0 (National Cancer Institute).^16,17^ Women mailed back their FFQ after completion. Study coordinators contacted each participant to obtain any missing information. Records with implausible dietary data (<600 or >6000 kcal per day) were excluded.

#### PHDI

The PHDI total and component scores were derived using a standardized scoring criteria.^18^ The PHDI total score is the sum of measures on indicators for 14 food components, gauged in accordance with the recommended ranges listed in the EAT-*Lancet* Commission Scientific Report; this approach has been validated.^6,19^ The total score has a minimum possible value of 0 and maximum value of 140. Dietary consumption between the minimum and maximum were scored proportionately (eTable 1 in Supplement 1).

#### Fetal Growth Outcomes

Fetal growth was measured up to 5 times between 15 to 42 weeks’ gestation, with ultrasonography visits scheduled at regular intervals across pregnancy. At each visit, 2D biometric measurements and 3D volumes were obtained using standard operating procedures and identical equipment (Voluson E8 [GE Healthcare]). Drawing on these data, our analysis included the following fetal measurements: HC; humerus length (HL); abdominal circumference (AC); femur length (FL); fractional arm volumes (total, lean, and fat); fractional thigh volumes (total, lean, and fat); maximum arm, abdominal, and thigh subcutaneous tissue thickness (SCTT); abdominal area; and organ volumes (ie, cerebellum, lung, kidney, and liver).

In conjunction with data collection, quality assurance was performed on a random sample of 10% of the ultrasonography images with generally good intrarater and interrater agreement.^12^ Further details regarding study protocol have been published elsewhere.^12,13,14,20,21^ Estimated fetal weight (EFW) was calculated from HC, AC, and FL using a Hadlock formula.^22^

### Covariates

At time of enrollment, research staff conducted interviews with study participants to collect covariates: maternal age, self-reported race and ethnicity (Asian or Pacific Islander, Hispanic, non-Hispanic Black, and non-Hispanic White), education (less than high school, high school or General Educational Development, some college, college, or advanced degree), marital status (married, living as married, or not married), job status (yes or no), prepregnancy BMI (<24.9, 25.0-29.9, and ≥30.0) based on self-reported prepregnancy weight and measured height, and parity (0, 1, and ≥2). Information on infant sex (male and female) was obtained from delivery medical records.

### Statistical Analysis

Data analysis was performed from June 2024 to August 2025. Descriptive statistics of maternal and child characteristics were compared using analysis of variance for continuous variables and χ^2^ tests for categorical variables. Given lack of established thresholds for the PHDI, participants were classified into tertiles based on their PHDI scores, consistent with current literature.^18,23,24^ All fetal 2D and 3D biometrics were log-transformed to approximate a normal distribution prior to analyses. Model assumptions were evaluated by examining residual plots for normality, homoscedasticity, and independence; no major violations were observed. To calculate trajectories of fetal 3D body composition, linear-mixed models were fit using restricted maximum likelihood. The model included fixed-effects of linear, quadratic, and cubic spline terms of gestational age (3 knots at the 25th, 50th, and 75th percentiles), as well as covariates listed previously. A random intercept and slope for gestational age was included with an unstructured covariance to account for correlations among repeated ultrasonography measurements within each fetus. To evaluate overall differences in fetal growth trajectories across PHDI tertiles, the global likelihood ratio test compared (1) a model with all covariates, including PHDI tertiles and their interaction with gestational age terms and (2) a model with only covariates. Weekly marginal means were estimated between 15 to 40 weeks’ gestation in the R lsmeans package, with *P* values based on Wald tests. All means and confidence intervals were back-transformed.

In sensitivity analyses, data were restricted to women in the standard population—without obese prepregnancy BMI and without a pregnancy complication (gestational diabetes, gestational hypertension, preeclampsia, or preterm birth)—to assess whether associations of PHDI with fetal growth were independent of complications. Tukey multiple comparison correction assessed robustness. All analyses were performed using SAS version 9.4 (SAS) or R version 4.2.2 (R Project for Statistical Computing) and had statistical significance established at an α level of .05 (2-tailed).

## Results

**Figure 1.**
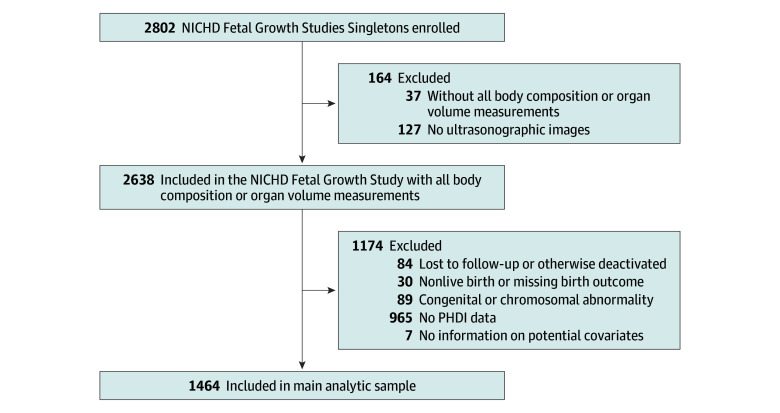
Flowchart for Inclusion of Study Participants in the National Institute of Child Health and Human Development (NICHD) Fetal Growth Studies—Singletons, 2015-2019 PHDI indicates Planetary Health Diet Index.

The final sample included 1464 women (273 Asian [18.7%]; 447 Hispanic [30.5%]; 433 non-Hispanic Black [29.6%]; and 311 non-Hispanic White [21.2%]), with a mean (SD) maternal age of 28.1 (5.6) years and mean (SD) gestational age at delivery of 39.2 (1.7) weeks (Figure 1). The baseline characteristics of participants across PHDI tertiles are presented in Table 1. Women were categorized into tertiles of PHDI adherence (tertile 1 [low; PHDI, ≤83]: 480 women; tertile 2 [moderate; PHDI, 84-92]: 491 women; tertile 3 [high; PHDI, ≥93]: 493 women). Study participants with low PHDI adherence were more likely to be of non-Hispanic Black race and ethnicity, younger, have a higher prepregnancy BMI, and less likely to have an advanced college degree compared with those with moderate or high PHDI (Table 1).

**Table 1.** Study Sample Characteristics Across PHDI: National Institute of Child Health and Human Development Fetal Growth Studies—Singletons, 2009-2013

Characteristic	Participants by PHDI adherence, No. (%)				*P* value^a^
	Overall (N = 1464)	Tertile 1: low (n = 480)	Tertile 2: moderate (n = 491)	Tertile 3: high (n = 493)	
PHDI total score, mean (SD)	76.2 (6.0)	76.2 (6.0)	87.8 (2.7)	98.7 (4.9)	.49
Maternal age, mean (SD) y	28.1 (5.6)	26.4 (5.6)	28.4 (5.6)	29.6 (5.1)	<.001
Maternal race and ethnicity					
Asian or Pacific Islander	273 (18.7)	57 (11.9)	95 (19.4)	121 (24.5)	<.001
Hispanic	447 (30.5)	122 (25.4)	182 (37.1)	143 (29.0)	
Non-Hispanic Black	433 (29.6)	243 (50.6)	112 (22.8)	78 (15.8)	
Non-Hispanic White	311 (21.2)	58 (12.1)	102 (20.8)	151 (30.6)	
Maternal education					
Less than high school	167 (11.4)	71 (14.8)	62 (12.6)	34 (6.9)	<.001
High school or equivalent	287 (19.6)	120 (25.0)	95 (19.4)	72 (14.6)	
Some college or Associate’s degree	456 (31.2)	168 (35.0)	157 (32.0)	131 (26.6)	
Bachelor’s degree	321 (21.9)	82 (17.1)	105 (21.4)	134 (27.2)	
Advanced degree	233 (15.9)	39 (8.1)	72 (14.7)	122 (24.8)	
Married	1070 (73.1)	285 (59.4)	379 (77.2)	406 (82.4)	<.001
Job status	1017 (69.5)	338 (70.4)	330 (67.2)	349 (70.8)	.41
Prepregnancy BMI^b^					
Mean (SD)	25.4 (5.1)	26.0 (5.3)	25.5 (5.1)	24.8 (4.9)	.001
Normal weight (<24.9)	1356 (55.9)	249 (51.9)	276 (56.2)	298 (60.5)	.05
Overweight (25.0-29.9)	664 (27.4)	135 (28.1)	129 (26.3)	128 (26.0)	
Obese (≥30.0)	407 (16.8)	96 (20.0)	86 (17.5)	67 (13.6)	
Parity					
0	671 (45.8)	221 (46.0)	210 (42.8)	240 (48.7)	.19
1	510 (34.8)	162 (33.8)	175 (35.6)	173 (35.1)	
≥2	283 (19.3)	97 (20.2)	106 (22.0)	80 (16.2)	
Gestational age at delivery, mean (SD), wk	39.2 (1.7)	39.2 (1.8)	39.1 (1.8)	39.4 (1.5)	.13
Infant sex					
Male	735 (50.2)	233 (48.5)	233 (47.5)	269 (54.6)	.06
Female	729 (49.8)	247 (51.5)	258 (52.6)	224 (45.4)	

Abbreviations: BMI, body mass index; PHDI, Planetary Health Diet Index.

^a^ *P* values were obtained from comparisons between tertiles using analysis of variance for continuous variables and χ^2^ for categorical variables.

^b^ Calculated as weight in kilograms divided by height in meters squared.

### Fetal Body Composition Volumes

Both high and moderate PHDI adherence were significantly associated with differences in fetal body composition volumes, relative to women with low PHDI adherence (Table 2). Specifically, results of our analysis indicated fetuses of women with high PHDI adherence had larger mean EFW by 35 g (95% CI, 22-49 g) starting at 32 weeks and persisting through 40 weeks’ gestation, with differences increasing to 165 g (95% CI, 108-223 g). Mean HC demonstrated significant differences across 37 to 39 weeks’ gestation, being 1.89 mm (95% CI, 1.10-2.69 mm) larger at 37 weeks and increasing to 2.44 mm (95% CI, 1.47-3.42 mm) at 39 weeks, along with mean FL that was 0.39 mm (95% CI, 0.23-0.54 mm) larger at 15 weeks and 1.18 mm (95% CI, 0.69-1.66 mm) larger at 40 weeks.

**Table 2.** Global and Weekly Comparisons of Longitudinal, 2-Dimensional and 3-Dimensional Fetal Growth, Body Composition, and Organ Volumes Across Planetary Health Diet Index Tertiles: National Institute of Child Health and Human Development Fetal Growth Studies—Singletons^a^

Outcome	Participants, No. (N = 1464)	Trajectory comparison *P* value^b^	Weekly comparisons			
			Tertile 2 vs tertile 1		Tertile 3 vs tertile 1	
			GA, wk	Magnitude of association	GA, wk	Magnitude of association
Body composition						
Estimated fetal weight, g	1443	.01	32-39	Larger	32-40	Larger
2-Dimensional measures						
Head circumference, mm	1449	.04	37-38	Larger	37-39	Larger
Abdominal circumference, mm	1448	.30	30-36	Larger	26-36	Larger
Humerus length, mean (SD), mm	1449	.04	15-16	Larger	19-21	Smaller
Femur length, mean (SD) mm	1450	.02	15-16; 40	Larger; larger	15, 40	Larger; larger
3-Dimensional measures						
Fractional arm volume, cm^3^	915	.74	NA^c^	NA^c^	NA^c^	NA^c^
Fractional lean arm volume, cm^3^	775	.21	34-38	Smaller	34-37	Smaller
Fractional fat arm volume, cm^3^	775	.35	35-38	Larger	28-29; 36-38	Larger; larger
Maximum arm SCTT, cm	846	.61	NA^c^	NA^c^	NA^c^	NA^c^
Abdominal area, mm^2^	1360	.16	15-16; 26-27; 39-40	Larger; larger; larger	15; 25-26	Larger; larger
Maximum abdominal SCTT, cm	1290	.43	21-32; 36-38	Larger; larger	NA^c^	NA^c^
Fractional thigh volume, cm^3^	910	.56	NA^c^	NA^c^	NA^c^	NA^c^
Fractional lean thigh volume, cm^3^	801	.39	37-38	Smaller	NA^c^	NA^c^
Fractional fat thigh volume, cm^3^	800	.64	NA^c^	NA^c^	NA^c^	NA^c^
Maximum thigh SCTT, cm	844	.65	NA^c^	NA^c^	NA^c^	NA^c^
Organ volumes						
Cerebellar volume, cm^3^	772	.64	NA^c^	NA^c^	NA^c^	NA^c^
Average Lung volume, mean (SD), cm^3^	128	.50	26-29	Larger	NA^c^	NA^c^
Kidney volume, mean (SD), cm^3^	540	.35	15-16; 22-25	Larger; Larger	NA^c^	NA^c^
Liver volume, cm^3^	652	.74	NA^c^	NA^c^	NA^c^	NA^c^

Abbreviations: GA, gestational age; NA, not applicable; SCTT, subcutaneous tissue thickness.

^a^ Linear mixed models were adjusted for maternal age, race and ethnicity, parity, prepregnancy body mass index, education, infant sex, job status, and married status. The eTables used for construction of this Table were eTables 2 to 20 in Supplement 1.

^b^ Reported *P* values are uncorrected for multiple comparisons.

^c^ No gestational age for which pairwise comparisons were different.

**Figure 2.**
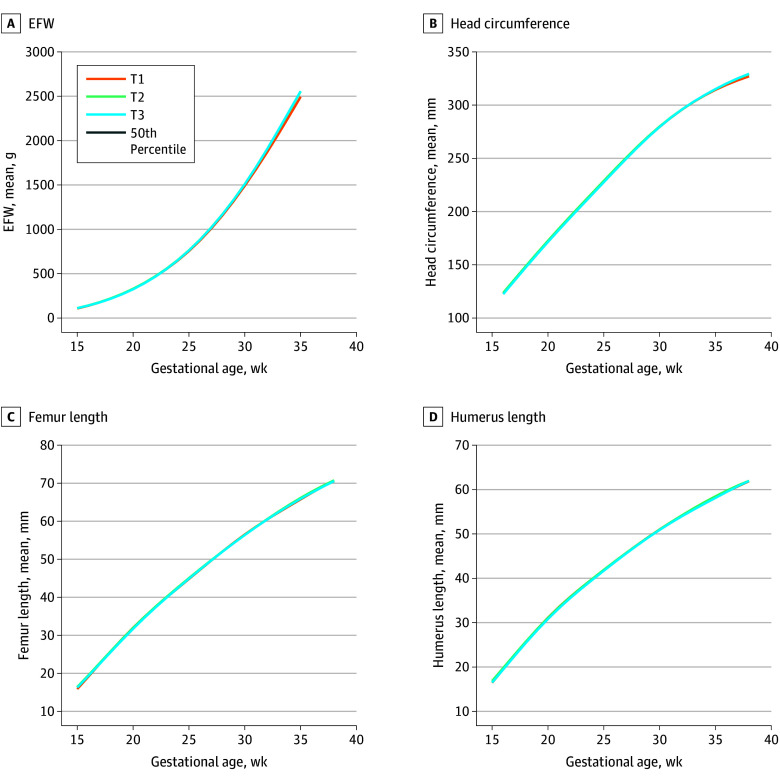
Two- and 3-Dimensional Fetal Body Composition Trajectories by Gestational Age Across Planetary Health Diet Index Tertiles (T): National Institute of Child Health and Human Development Fetal Growth Studies—Singletons, 2015-2019 Trajectory graph displays growth from 15 to 35 weeks’ gestational age for estimated fetal weight (EFW; A), 16 to 38 weeks’ gestational age for head circumference (B), 15 to 38 weeks’ gestational age for femur length (C), and 15 to 38 weeks' for humerus length (D).

For fetuses of women with moderate PHDI adherence, results were similar. EFW was larger between 32 to 39 weeks, starting at 33 g (95% CI, 20 to 47 g) at 32 weeks and increasing to 90 g (95% CI, 58 to 123 g) by 39 weeks. HC was larger at 37 weeks (mean difference, 1.71 mm; 95% CI, 0.91 to 2.51 mm), with the magnitude of difference increasing to 2.03 mm (95% CI, 1.18 to 2.88 mm) larger by 38 weeks. Between 15 to 16 weeks, mean FL was significantly larger (mean difference, 0.55 mm; 95% CI, 0.38 to 0.73 mm) at 15 weeks, remaining larger at 0.37 mm (95% CI, 0.24 to 0.50 mm) by 16 weeks (Table 2, Figure 2, and eTables 2-6 in Supplement 1). Although high PHDI adherence was associated with smaller mean HL at 19 weeks (mean difference, −0.26 mm; 95% CI, −0.38 to −0.14 mm), with differences increasing by 21 weeks (mean difference, −0.29 mm; 95% CI, −0.42 to −0.16 mm), HL was larger at 15 weeks (mean difference, 0.41 mm; 95% CI, 0.25 to 0.58 mm) and remaining larger with differences increasing to 0.29 mm (95% CI, 0.17 to 0.42 mm) by 16 weeks for fetuses of women with moderate adherence between 15 and 16 weeks.

### Fetal Body Composition Trajectories

Trajectories of fetal body composition during gestation showed limited variation across PHDI tertiles. Fetuses of women with high adherence to the PHDI had a 0.40 cm^3^ (95% CI, 0.25-0.56 cm^3^) smaller fractional lean arm volume starting at 34 weeks, increasing to 0.64 cm^3^ (95% CI, 0.36-0.92 cm^3^) smaller by 37 weeks. Mean fractional fat arm volume was 0.23 cm^3^ (95% CI, 0.13 to 0.34 cm^3^) larger at 28 weeks, increasing to 0.25 cm^3^ (95% CI, 0.13-0.36 cm^3^) by 29 weeks, and remaining larger between 36 to 38 weeks. Mean abdominal area was larger at 15 weeks (40.0 mm^2^; 95% CI, 23.6-56.3 mm^2^) and 62.2 mm^2^ (95% CI, 34.4 to 90.0 mm^2^) larger starting at 25 weeks, with mean differences increasing to 65.5 mm^2^ (95% CI, 36.4-94.5 mm^2^) by 26 weeks, compared with fetuses of women with low PHDI adherence (Table 2 and eTables 7-11 in Supplement 1).

Further, we observed that fetuses of women with moderate PHDI adherence relative to women with low adherence had 0.42 cm^3^ (95% CI, 0.26-0.58 cm^3^) smaller fractional lean arm volume starting at 34 weeks, with differences increasing to 0.92 cm^3^ (95% CI, 0.60-1.25 cm^3^) smaller by 38 weeks. Differences were observed in later gestation with a 253.4 mm^2^ (95% CI, 152.8-354.1 mm^2^) larger abdominal area starting at 39 weeks and increasing to 467.1 mm^2^ (95% CI, 263.2- 671.0 mm^2^) larger by 40 weeks. Similarly, maximum abdominal SCTT was significantly larger starting at 36 weeks (0.23 cm; 95% CI, 0.13-0.32 cm), increasing to 0.32 cm (95% CI, 0.20-0.44 cm) larger by 38 weeks. Lastly, fractional lean thigh volume was 2.19 cm^3^ (95% CI, 1.35-3.03 cm^3^) smaller at 37 weeks and increased in difference to 2.71 cm^3^ (95% CI, 1.73-3.69 cm^3^) smaller by 38 weeks in fetuses of women with high PHDI adherence compared with women with low adherence (Table 2; eTables 8 and 11-16 in Supplement 1).

### Organ Volumes

Fetal organ volumes among women with high PHDI adherence did not differ significantly on a week-by-week basis for fetuses of women with low adherence. Our analysis indicated select differences among fetuses of women with moderate PHDI adherence. Mean lung volume was 2.77 mm^2^ (95% CI, 1.55-3.99 mm^2^) larger starting at 26 weeks and increasing in difference to 3.89 mm^2^ (95% CI, 2.01-5.77 mm^2^) larger by 29 weeks, while mean kidney volume was significantly larger starting at 15 weeks (mean difference, 0.20 mm^2^; 95% CI, 0.10-0.30 mm^2^), with the mean difference smaller by 16 weeks (mean difference, 0.15 mm^2^; 95% CI, 0.09-0.22 mm^2^) and with a mean difference ranging from 0.21 mm^2^ (95% CI, 0.11-0.31 mm^2^) to 0.43 mm^2^ (95% CI, 0.26-0.59 mm^2^) larger between 22 and 25 weeks (Table 2; eTables 17-20 in Supplement 1).

### Sensitivity Analyses

When we conducted analyses restricting the sample to the standard population (1034 women), findings remained consistent with the main analysis, although the overall differences were attenuated in EFW, FL, and HL (Table 3 and eTables 21-25 in Supplement 1). For weekly comparisons of 3D measures, differences were generally consistent with the main analysis. Contrary to the main analysis, where we observed no differences in fractional fat thigh volume across all levels of PHDI adherence, women with high and moderate adherence had smaller fractional fat thigh volume between 18 to 21 weeks compared with those with low adherence (high adherence: 0.10 cm^3^; 95% CI, 0.06-0.14 cm^3 ^ to 16.00 cm^3^; 95% CI, 0.09 to 0.23 cm^3 ^smaller; moderate adherence: 0.10 cm^3^; 95% CI, 0.06-0.14 cm^3^ to 0.15 cm^3^; 95% CI, 0.08-0.22 cm^3 ^smaller) (eTables 26-35 in Supplement 1). No consistent weekly associations were observed for fetal organ volume trajectories across PHDI tertiles, except for larger liver volume between 32 weeks (6.93 mm^2^; 95% CI, 3.88-9.97 mm^2^) to 34 weeks (8.22 mm^2^; 95% CI, 4.60-11.84 mm^2^) among fetuses of women with moderate PHDI adherence (eTables 36-39 in Supplement 1). Associations between fetal ultrasonography measures across PHDI tertiles were generally unchanged after assessment using Tukey method for multiple comparison correction in the standard population group (eTable 40 [uncorrected for multiple comparisons] and eTable 41 [corrected] in Supplement 1).

**Table 3.** Global and Weekly Comparisons of Longitudinal, 2-Dimensional and 3-Dimensional Fetal Growth, Body Composition, and Organ Volumes Across Planetary Health Diet Index Tertiles Among the Standard Population: National Institute of Child Health and Human Development Fetal Growth Studies—Singletons^a^

Outcome	Participants, No. (N = 1034)^b^	Global comparison *P* value^c^	Weekly comparisons			
			Tertile 2 vs tertile 1		Tertile 3 vs tertile 1	
			GA, wk	Magnitude of association	GA, wk	Magnitude of association
Body composition						
Estimated fetal weight, g	1022	.08	32-39	Larger	33-39	Larger
2-Dimensional measures						
Head circumference, mm	1025	.10	38	Larger	NA^d^	NA^d^
Abdominal circumference, mm	1025	.61	31-37	Larger	33-36	Larger
Humerus length, mean (SD), mm	1026	.19	NA^d^	NA^d^	NA^d^	NA^d^
Femur length, mean (SD), mm	1024	.21	NA^d^	NA^d^	17-23	Smaller
3-Dimensional measures						
Fractional arm volume, cm^3^	675	.66	NA^d^	NA^d^	NA^d^	NA^d^
Fractional lean arm volume, cm^3^	576	.78	NA^d^	NA^d^	NA^d^	NA^d^
Fractional fat arm volume, cm^3^	576	.22	35-38	Larger	28-29	Larger
Maximum arm SCTT, cm	621	.74	NA^d^	NA^d^	NA^d^	NA^d^
Abdominal area, mm^2^	957	.29	34-35, 39-40	Larger, larger	NA^d^	NA^d^
Maximum abdominal SCTT, cm	913	.07	21-35, 39-40	Larger, larger	40	Smaller
Fractional thigh volume, cm^3^	674	.41	NA^d^	NA^d^	NA^d^	NA^d^
Fractional lean thigh volume, cm^3^	594	.85	NA^d^	NA^d^	NA^d^	NA^d^
Fractional fat thigh volume, cm^3^	593	.23	18-21	Smaller	18-21	Smaller
Maximum thigh SCTT, cm	621	.62	NA^d^	NA^d^	NA^d^	NA^d^
Organ volumes						
Cerebellar volume, cm^3^	566	.21	NA^d^	NA^d^	NA^d^	NA^d^
Lung volume, mean (SD), cm^3^	94	.67	NA^d^	NA^d^	NA^d^	NA^d^
Kidney volume, mean (SD), cm^3^	225	.72	NA^d^	NA^d^	NA^d^	NA^d^
Liver volume, cm^3^	479	.65	32-34	Larger	NA^d^	NA^d^

Abbreviations: GA, gestational age; NA, not applicable; SCTT, subcutaneous tissue thickness.

^a^ Linear mixed models were adjusted for maternal age, race and ethnicity, parity, prepregnancy body mass index, education, infant sex, job status, and married status. The eTables used to construct this Table were those involving the Standard Population and include eTables 21 to 40 in Supplement 1.

^b^ Women without an obese prepregnancy body mass index and without a pregnancy complication in the current pregnancy.

^c^ Reported *P* values are uncorrected for multiple comparisons.

^d^ No gestational age for which pairwise comparisons were different.

## Discussion

In this longitudinal cohort study of pregnant women, we found that maternal adherence to the PHD was associated with significant differences in fetal body composition volumes and trajectories. Specifically, fetuses of women with high PHDI adherence exhibited larger EFW and HC within the third trimester, smaller mean HL in the second trimester, larger FL within the second trimester and across gestation, and larger abdominal area and larger fractional fat arm volume within the third trimester. Fetuses of women with moderate PHDI adherence had larger HL, mean lung volume, and mean kidney volume in the second trimester compared with those with high adherence.

Our findings are novel given the lack of studies examining the PHDI and fetal growth measures by 2D or 3D ultrasonography. The few studies that have examined adherence to other health indices and dietary patterns in relation to 2D fetal measures have yielded mixed results. Consistent with our findings, a study among 3207 non-Hispanic White pregnant women found low adherence to the Mediterranean diet was associated with lower EFW (adjusted β =  −0.11; 95% CI, −0.20 to −0.02) in the second trimester.^25^ However, 3 studies examining maternal dietary patterns found no association of adherence to dietary patterns with EFW^4,5,26^ or AC throughout the second and third trimester.^4,5^

Qin et al^4^ found mothers consuming meats and less nuts had fetuses with higher HC Z scores (β = 0.04; 95% CI, 0.02 to 0.07) across pregnancy, but smaller HC Z scores among mothers having higher consumption of vegetables and fish (β = −0.09; 95% CI, −0.12 to −0.06) in the second trimester. The latter diet is not identical to the PHD, but contrasts with our findings of larger HC in the third trimester among fetuses of women with moderate or high adherence to the PHDI. Therefore, these findings suggest that variation among diets in terms of the precise composition of plant-based products and animal-based products may be associated with differences in body composition volumes.

To our knowledge, only 2 studies have examined maternal dietary patterns and fetal 3D ultrasonography measures. One study of 228 pregnant women found a diet low in animal-based products but high in fish and olive oil was associated with accelerated embryonic development (β = 0.12; 95% CI, 0.00-0.24).^27^ Another study of 126 women reported high adherence to a dairy-rich pattern was associated with larger 3D fetal cerebellar volume (β = 0.02; 95% CI, 0.01-0.03).^28^ In contrast, our study found no significant associations of PHD adherence with fetal cerebellar volume, despite the PHD permitting moderate dairy intake as well as fortified plant-based alternatives.^29^ Thus, underlying mechanisms between animal- and plant-based dietary sources and fetal brain development warrant further exploration. Although one prior study investigating maternal dietary patterns and 2D kidney volumes found no association of micro- or macronutrients or diet quality with combined kidney volume or combined kidney volume relative to EFW,^30^ to our knowledge, our finding of larger average 3D kidney volume in the second trimester offers new insights.

Studies evaluating the association of maternal dietary intake during pregnancy and offspring adiposity have produced mixed results. One study among 179 mothers found maternal intake of starch (eg, carbohydrate source) in the diet to be associated with a higher percentage of fetal abdominal fat (*P* = .02).^31^ This association is consistent with previous studies evaluating neonatal adiposity. Starling et al^32^ found that, among a sample of 764 pregnant women, maternal adherence to dietary patterns characterized by intake of eggs, potatoes, other starchy vegetables, and nonwhole grains was associated with greater neonatal adiposity (0.94%; 95% CI, 0.26%-1.61%). Another study in 222 pregnant women with obesity found high intake of carbohydrates during late pregnancy was associated with greater newborn fat mass (2.1%; 95% CI, 0.6%-3.7%).^33^ A study in 1040 pregnant women found higher maternal intake of either fat (β =  4.24; 95% CI, 1.39-7.09) or carbohydrate (β = 2.93; 95% CI, 0.46-5.40) was associated with offspring fat mass.^34^ These findings suggest greater plant-based intake with moderate animal protein may influence fetal fat deposition via maternal lipid metabolism, while lean tissue growth relying on protein and amino acids,^35,36^ supporting evidence that maternal dietary patterns and nutrient intake vary. Thus, our findings corroborate emerging evidence that suggests certain patterns of dietary consumption and variations in nutrient intake by the mother during pregnancy could contribute to in utero fetal body composition and newborn size.

### Strengths and Limitations

Among the strengths of our study is the utilization of data from a sample consisting of varying race ethnicity groups of women across the US, which may increase generalizability compared with prior studies. In addition, we conducted a detailed assessment of dietary intake using a validated instrument. Ultrasonography collection was conducted by trained sonographers at multiple points during pregnancy, which allowed for an accurate, robust estimation of fetal growth compared with other prospective studies that assessed fetal biometric measures in either early or mid- to late pregnancy. Our study sample also comprised lower-risk pregnancies, enabling the examination of differences in 3D fetal growth across categories of adherence to the PHDI with minimal influence from adverse pregnancy factors that could affect potential associations.

The source of the data used in our study has several potential limitations that have been previously mentioned.^13,15^ Our available sample with 3D fetal measures was smaller than our 2D sample, due to image quality that was appropriate for 2D vs 3D evaluation, which may affect our ability to detect differences. Therefore, our findings should be considered exploratory in nature. Lastly as an observational study, residual confounding remains a limitation.

## Conclusions

In this cohort study, fetuses of women with high PHDI adherence had larger EFW, head circumference, fractional fat arm volume, and abdominal measures in the third trimester, with similar patterns for moderate adherence from the second trimester onward. Although some differences were statistically significant, they may not be clinically meaningful and are hypothesis-generating rather than indicative of clinical outcomes. While the PHDI may support environmental sustainability, its associations with fetal adiposity require further study. Future research should examine maternal PHDI adherence alongside other lifestyle factors to clarify fetal fat accumulation and potential associations with neonatal and childhood growth, including overweight and obesity risk.

## Notes

Supplement 1. eTable 1. Scoring Criteria for the Planetary Health Diet Index by Frank et al eTable 2. Means of Estimated Fetal Weight by Gestational Week in Fetuses of Women With PHDI (T1), PHDI (T2), and PHDI (T3) eTable 3. Means of Head Circumference by Gestational Week in Fetuses of Women With PHDI (T1), PHDI (T2), and PHDI (T3) eTable 4. Means of Abdominal Circumference by Gestational Week in Fetuses of Women With PHDI (T1), PHDI (T2), and PHDI (T3) eTable 5. Means of Average Humerus Length by Gestational Week in Fetuses of Women With PHDI (T1), PHDI (T2), and PHDI (T3) eTable 6. Means of Average Femur Length by Gestational Week in Fetuses of Women With PHDI (T1), PHDI (T2), and PHDI (T3) eTable 7. Means of Fractional Arm Volume by Gestational Week in Fetuses of Women With PHDI (T1), PHDI (T2), and PHDI (T3) eTable 8. Means of Fractional Lean Arm Volume by Gestational Week in Fetuses of Women With PHDI (T1), PHDI (T2), and PHDI (T3) eTable 9. Means of Fractional Fat Arm Volume by Gestational Week in Fetuses of Women With PHDI (T1), PHDI (T2), and PHDI (T3) eTable 10. Means of Maximum Arm Subcutaneous Tissue Thickness (SCTT) by Gestational Week in Fetuses of Women With PHDI (T1), PHDI (T2), and PHDI (T3) eTable 11. Means of Abdominal Area by Gestational Week in Fetuses of Women With PHDI (T1), PHDI (T2), and PHDI (T3) eTable 12. Means of Maximum Abdominal Subcutaneous Tissue Thickness (SCTT) by Gestational Week in Fetuses of Women With PHDI (T1), PHDI (T2), and PHDI (T3) eTable 13. Means of Fractional Thigh Volume by Gestational Week in Fetuses of Women With PHDI (T1), PHDI (T2), and PHDI (T3) eTable 14. Means of Fractional Lean Thigh Volume by Gestational Week in Fetuses of Women With PHDI (T1), PHDI (T2), and PHDI (T3) eTable 15. Means of Fractional Fat Thigh Volume by Gestational Week in Fetuses of Women With PHDI (T1), PHDI (T2), and PHDI (T3) eTable 16. Means of Maximum Thigh Subcutaneous Tissue Thickness (SCTT) by Gestational Week in Fetuses of Women With PHDI (T1), PHDI (T2), and PHDI (T3) eTable 17. Means of Cerebellar Volume by Gestational Week in Fetuses of Women With PHDI (T1), PHDI (T2), and PHDI (T3) eTable 18. Means of Average Lung Volume by Gestational Week in Fetuses of Women With PHDI (T1), PHDI (T2), and PHDI (T3) eTable 19. Means of Kidney by Gestational Week in Fetuses of Women With PHDI (T1), PHDI (T2), and PHDI (T3) eTable 20. Means of Liver by Gestational Week in Fetuses of Women With PHDI (T1), PHDI (T2), and PHDI (T3) eTable 21. Means of Estimated Fetal Weight by Gestational Week in Fetuses of Women With PHDI (T1), PHDI (T2), and PHDI (T3) Among the Standard Population eTable 22. Means of Head Circumference by Gestational Week in Fetuses of Women With PHDI (T1), PHDI (T2), and PHDI (T3) among the Standard Population eTable 23. Means of Abdominal Circumference by Gestational Week in Fetuses of Women With PHDI (T1), PHDI (T2), and PHDI (T3) among the Standard Population eTable 24. Means of Average Humerus Length by Gestational Week in Fetuses of Women with PHDI (T1), PHDI (T2), and PHDI (T3) Among the Standard Population eTable 25. Means of Average Femur Length by Gestational Week in Fetuses of Women With PHDI (T1), PHDI (T2), and PHDI (T3) Among the Standard Population eTable 26. Means of Fractional Arm Volume by Gestational Week in Fetuses of Women With PHDI (T1), PHDI (T2), and PHDI (T3) Among the Standard Population eTable 27. Means of Fractional Lean Arm Volume by Gestational Week in Fetuses of Women With PHDI (T1), PHDI (T2), and PHDI (T3) Among the Standard Population eTable 28. Means of Fractional Fat Arm Volume by Gestational Week in Fetuses of Women With PHDI (T1), PHDI (T2), and PHDI (T3) Among the Standard Population eTable 29. Means of Maximum Arm Subcutaneous Tissue Thickness (SCTT) by Gestational Week in Fetuses of Women With PHDI (T1), PHDI (T2), and PHDI (T3) Among the Standard Population eTable 30. Means of Abdominal Area by Gestational Week in Fetuses of Women With PHDI (T1), PHDI (T2), and PHDI (T3) Among the Standard Population eTable 31. Means of Maximum Abdominal Subcutaneous Tissue Thickness (SCTT) by Gestational Week in Fetuses of Women With PHDI (T1), PHDI (T2), and PHDI (T3) Among the Standard Population eTable 32. Means of Fractional Thigh Volume by Gestational Week in Fetuses of Women With PHDI (T1), PHDI (T2), and PHDI (T3) Among the Standard Population eTable 33. Means of Fractional Lean Thigh Volume by Gestational Week in Fetuses of Women With PHDI (T1), PHDI (T2), and PHDI (T3) Among the Standard Population eTable 34. Means of Fractional Fat Thigh Volume by Gestational Week in Fetuses of Women With PHDI (T1), PHDI (T2), and PHDI (T3) Among the Standard Population eTable 35. Means of Maximum Thigh Subcutaneous Tissue Thickness (SCTT) by Gestational Week in Fetuses of Women With PHDI (T1), PHDI (T2), and PHDI (T3) Among the Standard Population eTable 36. Means of Cerebellar Volume by Gestational Week in Fetuses of Women With PHDI (T1), PHDI (T2), and PHDI (T3) Among the Standard Population eTable 37. Means of Average Lung Volume by Gestational Week in Fetuses of Women With PHDI (T1), PHDI (T2), and PHDI (T3) Among the Standard Population eTable 38. Means of Kidney by Gestational Week in Fetuses of Women With PHDI (T1), PHDI (T2), and PHDI (T3) Among the Standard Population eTable 39. Means of Liver by Gestational Week in Fetuses of Women With PHDI (T1), PHDI (T2), and PHDI (T3) Among the Standard Population. eTable 40. Global and Weekly Comparisons of Longitudinal, Two-Dimensional and Three-Dimensional Fetal Growth, Body Composition, and Organ Volumes Across PHDI Tertiles Among the Standard Population; NICHD Fetal Growth Studies eTable 41. Global and Weekly Comparisons of Longitudinal, Two-Dimensional and Three-Dimensional Fetal Growth, Body Composition, and Organ Volumes Across PHDI Tertiles Correcting for Multiple Comparisons; NICHD Fetal Growth Studies-Singletons

Supplement 2. Data Sharing Statement
