# Supplementary material for: Adherence to the Planetary Health Diet Index and Fetal Body Composition
Source: JAMA Netw Open. 2025 Dec 17;8(12):e2544153. doi: 10.1001/jamanetworkopen.2025.44153 (PMC12712731; doi:10.1001/jamanetworkopen.2025.44153)
Supplement: Supplement 1. — eTable 1. Scoring Criteria for the Planetary Health Diet Index by Frank et al eTable 2. Means of Estimated Fetal Weight by Gestational Week in Fetuses of Women With PHDI (T1), PHDI (T2), and PHDI (T3) eTable 3. Means of Head Circumference by Gestational Week in Fetuses of Women With PHDI (T1), PHDI (T2), and PHDI (T3) eTable 4. Means of Abdominal Circumference by Gestational Week in Fetuses of Women With PHDI (T1), PHDI (T2), and PHDI (T3) eTable 5. Means of Average Humerus Length by Gestational Week in Fetuses of Women With PHDI (T1), PHDI (T2), and PHDI (T3) eTable 6. Means of Average Femur Length by Gestational Week in Fetuses of Women With PHDI (T1), PHDI (T2), and PHDI (T3) eTable 7. Means of Fractional Arm Volume by Gestational Week in Fetuses of Women With PHDI (T1), PHDI (T2), and PHDI (T3) eTable 8. Means of Fractional Lean Arm Volume by Gestational Week in Fetuses of Women With PHDI (T1), PHDI (T2), and PHDI (T3) eTable 9. Means of Fractional Fat Arm Volume by Gestational Week in Fetuses of Women With PHDI (T1), PHDI (T2), and PHDI (T3) eTable 10. Means of Maximum Arm Subcutaneous Tissue Thickness (SCTT) by Gestational Week in Fetuses of Women With PHDI (T1), PHDI (T2), and PHDI (T3) eTable 11. Means of Abdominal Area by Gestational Week in Fetuses of Women With PHDI (T1), PHDI (T2), and PHDI (T3) eTable 12. Means of Maximum Abdominal Subcutaneous Tissue Thickness (SCTT) by Gestational Week in Fetuses of Women With PHDI (T1), PHDI (T2), and PHDI (T3) eTable 13. Means of Fractional Thigh Volume by Gestational Week in Fetuses of Women With PHDI (T1), PHDI (T2), and PHDI (T3) eTable 14. Means of Fractional Lean Thigh Volume by Gestational Week in Fetuses of Women With PHDI (T1), PHDI (T2), and PHDI (T3) eTable 15. Means of Fractional Fat Thigh Volume by Gestational Week in Fetuses of Women With PHDI (T1), PHDI (T2), and PHDI (T3) eTable 16. Means of Maximum Thigh Subcutaneous Tissue Thickness (SCTT) by Gestational Week in Fetuses of Women With PHDI (T1), PHDI [file jamanetwopen-e2544153-s001.pdf]

# Supplemental Online Content

Clayton PK, DeVilbiss EA, Lim SX, et al. Adherence to the Planetary Health Diet Index and Fetal Body Composition. *JAMA Netw. Open*. 2025;8(11):e2544153. doi:10.1001/jamanetworkopen.2025.44153

**eTable 1.** Scoring Criteria for the Planetary Health Diet Index by Frank et al

**eTable 2.** Means of Estimated Fetal Weight by Gestational Week in Fetuses of Women With PHDI (T1), PHDI (T2), and PHDI (T3)

**eTable 3.** Means of Head Circumference by Gestational Week in Fetuses of Women With PHDI (T1), PHDI (T2), and PHDI (T3)

**eTable 4.** Means of Abdominal Circumference by Gestational Week in Fetuses of Women With PHDI (T1), PHDI (T2), and PHDI (T3)

**eTable 5.** Means of Average Humerus Length by Gestational Week in Fetuses of Women With PHDI (T1), PHDI (T2), and PHDI (T3)

**eTable 6.** Means of Average Femur Length by Gestational Week in Fetuses of Women With PHDI (T1), PHDI (T2), and PHDI (T3)

**eTable 7.** Means of Fractional Arm Volume by Gestational Week in Fetuses of Women With PHDI (T1), PHDI (T2), and PHDI (T3)

**eTable 8.** Means of Fractional Lean Arm Volume by Gestational Week in Fetuses of Women With PHDI (T1), PHDI (T2), and PHDI (T3)

**eTable 9.** Means of Fractional Fat Arm Volume by Gestational Week in Fetuses of Women With PHDI (T1), PHDI (T2), and PHDI (T3)

**eTable 10.** Means of Maximum Arm Subcutaneous Tissue Thickness (SCTT) by Gestational Week in Fetuses of Women With PHDI (T1), PHDI (T2), and PHDI (T3)

**eTable 11.** Means of Abdominal Area by Gestational Week in Fetuses of Women With PHDI (T1), PHDI (T2), and PHDI (T3)

**eTable 12.** Means of Maximum Abdominal Subcutaneous Tissue Thickness (SCTT) by Gestational Week in Fetuses of Women With PHDI (T1), PHDI (T2), and PHDI (T3)

**eTable 13.** Means of Fractional Thigh Volume by Gestational Week in Fetuses of Women With PHDI (T1), PHDI (T2), and PHDI (T3)

**eTable 14.** Means of Fractional Lean Thigh Volume by Gestational Week in Fetuses of Women With PHDI (T1), PHDI (T2), and PHDI (T3)

**eTable 15.** Means of Fractional Fat Thigh Volume by Gestational Week in Fetuses of Women With PHDI (T1), PHDI (T2), and PHDI (T3)

© 2025 Clayton PK et al. *JAMA Network Open*.

**eTable 16.** Means of Maximum Thigh Subcutaneous Tissue Thickness (SCTT) by Gestational Week in Fetuses of Women With PHDI (T1), PHDI (T2), and PHDI (T3)

**eTable 17.** Means of Cerebellar Volume by Gestational Week in Fetuses of Women With PHDI (T1), PHDI (T2), and PHDI (T3)

**eTable 18.** Means of Average Lung Volume by Gestational Week in Fetuses of Women With PHDI (T1), PHDI (T2), and PHDI (T3)

**eTable 19.** Means of Kidney by Gestational Week in Fetuses of Women With PHDI (T1), PHDI (T2), and PHDI (T3)

**eTable 20.** Means of Liver by Gestational Week in Fetuses of Women WithPHDI (T1), PHDI (T2), and PHDI (T3)

**eTable 21.** Means of Estimated Fetal Weight by Gestational Week in Fetuses of Women With PHDI (T1), PHDI (T2), and PHDI (T3) Among the Standard Population

**eTable 22.** Means of Head Circumference by Gestational Week in Fetuses of Women With PHDI (T1), PHDI (T2), and PHDI (T3) among the Standard Population

**eTable 23.** Means of Abdominal Circumference by Gestational Week in Fetuses of Women With PHDI (T1), PHDI (T2), and PHDI (T3) among the Standard Population

**eTable 24.** Means of Average Humerus Length by Gestational Week in Fetuses of Women with PHDI (T1), PHDI (T2), and PHDI (T3) Among the Standard Population

**eTable 25.** Means of Average Femur Length by Gestational Week in Fetuses of Women With PHDI (T1), PHDI (T2), and PHDI (T3) Among the Standard Population

**eTable 26.** Means of Fractional Arm Volume by Gestational Week in Fetuses of Women With PHDI (T1), PHDI (T2), and PHDI (T3) Among the Standard Population

**eTable 27.** Means of Fractional Lean Arm Volume by Gestational Week in Fetuses of Women With PHDI (T1), PHDI (T2), and PHDI (T3) Among the Standard Population

**eTable 28.** Means of Fractional Fat Arm Volume by Gestational Week in Fetuses of Women With PHDI (T1), PHDI (T2), and PHDI (T3) Among the Standard Population

**eTable 29.** Means of Maximum Arm Subcutaneous Tissue Thickness (SCTT) by Gestational Week in Fetuses of Women With PHDI (T1), PHDI (T2), and PHDI (T3) Among the Standard Population

**eTable 30.** Means of Abdominal Area by Gestational Week in Fetuses of Women With PHDI (T1), PHDI (T2), and PHDI (T3) Among the Standard Population

© 2025 Clayton PK et al. *JAMA Network Open*.

**eTable 31.** Means of Maximum Abdominal Subcutaneous Tissue Thickness (SCTT) by Gestational Week in Fetuses of Women With PHDI (T1), PHDI (T2), and PHDI (T3) Among the Standard Population

**eTable 32.** Means of Fractional Thigh Volume by Gestational Week in Fetuses of Women With PHDI (T1), PHDI (T2), and PHDI (T3) Among the Standard Population

**eTable 33.** Means of Fractional Lean Thigh Volume by Gestational Week in Fetuses of Women With PHDI (T1), PHDI (T2), and PHDI (T3) Among the Standard Population

**eTable 34.** Means of Fractional Fat Thigh Volume by Gestational Week in Fetuses of Women With PHDI (T1), PHDI (T2), and PHDI (T3) Among the Standard Population

**eTable 35.** Means of Maximum Thigh Subcutaneous Tissue Thickness (SCTT) by Gestational Week in Fetuses of Women With PHDI (T1), PHDI (T2), and PHDI (T3) Among the Standard Population

**eTable 36.** Means of Cerebellar Volume by Gestational Week in Fetuses of Women With PHDI (T1), PHDI (T2), and PHDI (T3) Among the Standard Population

**eTable 37.** Means of Average Lung Volume by Gestational Week in Fetuses of Women With PHDI (T1), PHDI (T2), and PHDI (T3) Among the Standard Population

**eTable 38.** Means of Kidney by Gestational Week in Fetuses of Women With PHDI (T1), PHDI (T2), and PHDI (T3) Among the Standard Population

**eTable 39.** Means of Liver by Gestational Week in Fetuses of Women With PHDI (T1), PHDI (T2), and PHDI (T3) Among the Standard Population.

**eTable 40.** Global and Weekly Comparisons of Longitudinal, Two-Dimensional and Three-Dimensional Fetal Growth, Body Composition, and Organ Volumes Across PHDI Tertiles Among the Standard Population; NICHD Fetal Growth Studies

**eTable 41.** Global and Weekly Comparisons of Longitudinal, Two-Dimensional and Three-Dimensional Fetal Growth, Body Composition, and Organ Volumes Across PHDI Tertiles Correcting for Multiple Comparisons; NICHD Fetal Growth Studies-Singletons

This supplemental material has been provided by the authors to give readers additional information about their work.

© 2025 Clayton PK et al. *JAMA Network Open*.

**eTable 1**. Scoring Criteria for the Planetary Health Diet Index Frank *et al*.

| Dietary component | Criteria for minimum score (0 points) | Criteria for maximum score (10 points) |
|---|---|---|
| **Adequacy components** | | |
| Whole grains | 0 g | ≥ 75 g |
| Whole fruits (excludes fruit juice) | 0 g | ≥ 200 g |
| Non-starchy vegetables | 0 g | ≥ 300 g |
| Nuts and seeds | 0 g | ≥ 50 g |
| Legumes[1] | | |
| Non-soy legumes | 0 g | 100 g |
| Soybean/ soy foods | 0 g | 50 g |
| Unsaturated oils | 0 % of total energy intake | ≥ 10 % of total energy intake |
| **Moderation components** | | |
| Starchy vegetables | ≥ 200 g | ≤ 50 g |
| Dairy | ≥ 1000 g | ≤ 250 g |
| Red and processed meat | ≥ 300 g | ≤ 14 g |
| Poultry | ≥ 58 g | ≤ 29 g |
| Eggs | ≥ 120 g | ≤ 12 g |
| Fish | ≥ 50 g | ≤ 15 g |
| Saturated oils | ≥ 21 % of total energy intake | ≤ 3.5 % of total energy intake |
| Added sugars | ≥ 25 % of total energy intake | ≤ 5 % of total energy intake |

[1] To calculate the legumes component, the non-soy and soy subcomponents were equally weighted at 0.5 as recommended by the EAT-Lancet Commission Scientific Report.

© 2025 Clayton PK et al. *JAMA Network Open*.

**eTable 2.** Means of Estimated Fetal Weight by Gestational Week in Fetuses of Women with PHDI (T1), PHDI (T2), and PHDI (T3) in the NICHD Fetal Growth Studies-Singletons, 2009-2013.

| | Estimated Fetal Weight, g (95% CI) | | |
|---|---|---|---|
| GA | T1 | T2 | T3 |
| 15 | 108 (105,111) | 112 (109,115) | 111 (109,114) |
| 16 | 139 (137,141) | 143 (140,145) | 141 (139,143) |
| 17 | 176 (174,178) | 179 (177,181) | 176 (174,178) |
| 18 | 220 (218,223) | 222 (219,224) | 218 (215,220) |
| 19 | 271 (268,274) | 272 (269,275) | 267 (264,270) |
| 20 | 329 (325,332) | 330 (326,333) | 324 (321,328) |
| 21 | 394 (390,398) | 396 (392,400) | 391 (387,395) |
| 22 | 469 (464,473) | 472 (467,476) | 467 (462,471) |
| 23 | 553 (547,558) | 557 (552,563) | 553 (548,559) |
| 24 | 648 (641,654) | 654 (648,661) | 651 (645,658) |
| 25 | 755 (747,762) | 763 (756,771) | 761 (754,768) |
| 26 | 875 (867,884) | 885 (877,894) | 884 (875,892) |
| 27 | 1009 (1000,1018) | 1021 (1012,1030) | 1019 (1010,1028) |
| 28 | 1157 (1146,1167) | 1170 (1160,1181) | 1168 (1158,1179) |
| 29 | 1317 (1305,1330) | 1333 (1321,1345) | 1331 (1319,1344) |
| 30 | 1491 (1476,1505) | 1510 (1496,1524) | 1509 (1495,1523) |
| 31 | 1675 (1659,1691) | 1700 (1685,1715) | 1700 (1684,1716) |
| 32 | 1869 (1851,1887) | 1902 (1885,1919) | 1904 (1887,1922) |
| 33 | 2071 (2051,2091) | 2113 (2094,2133) | 2118 (2098,2138) |
| 34 | 2280 (2257,2303) | 2332 (2309,2355) | 2337 (2314,2360) |
| 35 | 2493 (2468,2519) | 2553 (2528,2579) | 2557 (2532,2582) |
| 36 | 2709 (2681,2737) | 2774 (2747,2802) | 2773 (2745,2801) |
| 37 | 2924 (2892,2956) | 2993 (2962,3025) | 2986 (2955,3018) |
| 38 | 3132 (3097,3168) | 3208 (3173,3244) | 3203 (3168,3238) |
| 39 | 3330 (3287,3373) | 3420 (3379,3461) | 3430 (3389,3471) |
| 40 | 3511 (3433,3591) | 3628 (3559,3699) | 3676 (3609,3744) |

© 2025 Clayton PK et al. *JAMA Network Open*.

**eTable 3.** Means of Head Circumference by Gestational Week in Fetuses of Women with PHDI (T1), PHDI (T2), and PHDI (T3) in the NICHD Fetal Growth Studies-Singletons, 2009-2013.

| | Mean Head Circumference, mm (95% CI) | | |
|---|---|---|---|
| GA | T1 | T2 | T3 |
| 15 | 110.1 (109.0,111.2) | 111.5 (110.3,112.7) | 110.0 (109.0,111.0) |
| 16 | 122.9 (122.1,123.6) | 123.9 (123.0,124.7) | 122.5 (121.8,123.2) |
| 17 | 135.6 (135.0,136.2) | 136.3 (135.7,136.9) | 135.0 (134.5,135.6) |
| 18 | 148.2 (147.6,148.8) | 148.6 (148.0,149.2) | 147.4 (146.9,148.0) |
| 19 | 160.5 (159.8,161.1) | 160.7 (160.1,161.3) | 159.6 (159.0,160.2) |
| 20 | 172.5 (171.8,173.2) | 172.6 (172.0,173.3) | 171.5 (170.8,172.1) |
| 21 | 184.2 (183.5,184.8) | 184.2 (183.6,184.9) | 183.1 (182.4,183.7) |
| 22 | 195.5 (194.9,196.2) | 195.6 (195.0,196.3) | 194.4 (193.7,195.0) |
| 23 | 206.6 (205.9,207.3) | 206.8 (206.1,207.5) | 205.5 (204.8,206.2) |
| 24 | 217.6 (216.9,218.4) | 217.9 (217.1,218.6) | 216.5 (215.8,217.2) |
| 25 | 228.6 (227.8,229.3) | 228.9 (228.2,229.6) | 227.6 (226.9,228.3) |
| 26 | 239.4 (238.7,240.2) | 239.8 (239.1,240.6) | 238.7 (237.9,239.4) |
| 27 | 250.1 (249.4,250.9) | 250.6 (249.9,251.3) | 249.5 (248.8,250.3) |
| 28 | 260.5 (259.7,261.2) | 260.9 (260.2,261.7) | 260.1 (259.4,260.8) |
| 29 | 270.3 (269.5,271.1) | 270.8 (270.1,271.6) | 270.2 (269.4,270.9) |
| 30 | 279.6 (278.8,280.4) | 280.0 (279.2,280.8) | 279.6 (278.8,280.3) |
| 31 | 288.1 (287.3,288.9) | 288.4 (287.6,289.2) | 288.1 (287.4,288.9) |
| 32 | 295.8 (295.0,296.6) | 296.0 (295.2,296.8) | 295.9 (295.1,296.7) |
| 33 | 302.8 (301.9,303.6) | 302.9 (302.1,303.7) | 302.9 (302.1,303.8) |
| 34 | 308.9 (308.0,309.9) | 309.2 (308.3,310.1) | 309.3 (308.4,310.2) |
| 35 | 314.4 (313.4,315.3) | 314.9 (314.0,315.8) | 315.1 (314.2,316.0) |
| 36 | 319.1 (318.2,320.1) | 320.2 (319.3,321.2) | 320.4 (319.5,321.3) |
| 37 | 323.3 (322.2,324.3) | 325.0 (324.0,326.0) | 325.2 (324.2,326.2) |
| 38 | 327.0 (325.9,328.1) | 329.0 (328.0,330.1) | 329.4 (328.3,330.4) |
| 39 | 330.4 (329.1,331.7) | 332.2 (331.0,333.4) | 332.8 (331.7,334.0) |
| 40 | 333.6 (331.1,336.2) | 334.2 (332.1,336.3) | 335.5 (333.5,337.6) |

© 2025 Clayton PK et al. *JAMA Network Open*.

**eTable 4**. Means of Abdominal Circumference by Gestational Week in Fetuses of Women with PHDI (T1), PHDI (T2), and PHDI (T3) in the NICHD Fetal Growth Studies-Singletons, 2009-2013.

| | Mean Abdominal Circumference, mm (95% CI) | | |
|---|---|---|---|
| GA | T1 | T2 | T3 |
| 15 | 89.7 (88.5,91.0) | 91.3 (89.9,92.7) | 90.9 (89.8,92.1) |
| 16 | 101.9 (101.1,102.8) | 103.1 (102.2,104.1) | 102.5 (101.7,103.3) |
| 17 | 114.2 (113.5,114.8) | 115.1 (114.4,115.8) | 114.3 (113.6,114.9) |
| 18 | 126.4 (125.7,127.0) | 127.0 (126.3,127.7) | 126.1 (125.5,126.8) |
| 19 | 138.3 (137.6,139.1) | 138.8 (138.1,139.5) | 138.0 (137.3,138.8) |
| 20 | 150.0 (149.2,150.8) | 150.4 (149.6,151.1) | 149.8 (149.0,150.5) |
| 21 | 161.3 (160.5,162.1) | 161.7 (160.9,162.5) | 161.4 (160.6,162.1) |
| 22 | 172.3 (171.5,173.1) | 172.8 (172.0,173.6) | 172.7 (172.0,173.5) |
| 23 | 183.1 (182.2,184.0) | 183.7 (182.9,184.5) | 184.0 (183.1,184.8) |
| 24 | 193.7 (192.8,194.7) | 194.5 (193.6,195.4) | 195.0 (194.1,195.9) |
| 25 | 204.4 (203.4,205.4) | 205.4 (204.4,206.3) | 206.0 (205.0,206.9) |
| 26 | 215.2 (214.2,216.2) | 216.3 (215.3,217.3) | 216.9 (215.9,217.8) |
| 27 | 225.9 (224.9,226.9) | 227.2 (226.3,228.2) | 227.7 (226.7,228.7) |
| 28 | 236.7 (235.7,237.7) | 238.2 (237.2,239.2) | 238.5 (237.5,239.5) |
| 29 | 247.5 (246.4,248.6) | 249.2 (248.1,250.2) | 249.3 (248.2,250.4) |
| 30 | 258.3 (257.1,259.4) | 260.1 (259.0,261.3) | 260.2 (259.1,261.3) |
| 31 | 269 (267.9,270.2) | 271.1 (270.0,272.3) | 271.2 (270.1,272.4) |
| 32 | 279.8 (278.6,281.0) | 282 (280.9,283.2) | 282.3 (281.1,283.5) |
| 33 | 290.4 (289.1,291.7) | 292.8 (291.6,294.1) | 293.3 (292.0,294.6) |
| 34 | 300.8 (299.4,302.3) | 303.4 (302.0,304.8) | 304 (302.6,305.4) |
| 35 | 311.0 (309.5,312.5) | 313.6 (312.2,315.0) | 314.1 (312.7,315.6) |
| 36 | 320.8 (319.2,322.3) | 323.4 (321.9,324.9) | 323.6 (322.1,325.1) |
| 37 | 330.1 (328.4,331.8) | 332.8 (331.1,334.4) | 332.6 (330.9,334.2) |
| 38 | 339.0 (337.2,340.9) | 341.8 (340.1,343.6) | 341.4 (339.7,343.2) |
| 39 | 347.4 (345.3,349.6) | 350.6 (348.6,352.7) | 350.5 (348.6,352.5) |
| 40 | 355.3 (351.2,359.5) | 359.3 (355.7,363.0) | 360.4 (356.9,363.8) |

© 2025 Clayton PK et al. *JAMA Network Open*.

**eTable 5.** Means of Average Humerus Length by Gestational Week in Fetuses of Women with PHDI (T1), PHDI (T2), and PHDI (T3) in the NICHD Fetal Growth Studies-Singletons, 2009-2013.

| | Mean Humerus Length, mm (95% CI) | | |
|---|---|---|---|
| GA | T1 | T2 | T3 |
| 15 | 16.5 (16.3,16.7) | 16.9 (16.6,17.1) | 16.5 (16.3,16.7) |
| 16 | 19.5 (19.4,19.7) | 19.8 (19.6,20.0) | 19.5 (19.3,19.6) |
| 17 | 22.6 (22.5,22.7) | 22.8 (22.6,22.9) | 22.5 (22.3,22.6) |
| 18 | 25.6 (25.5,25.7) | 25.7 (25.5,25.8) | 25.4 (25.3,25.5) |
| 19 | 28.5 (28.3,28.6) | 28.5 (28.3,28.6) | 28.2 (28.0,28.3) |
| 20 | 31.1 (31.0,31.3) | 31.1 (30.9,31.2) | 30.8 (30.7,31.0) |
| 21 | 33.6 (33.4,33.7) | 33.5 (33.4,33.7) | 33.3 (33.1,33.4) |
| 22 | 35.8 (35.7,36.0) | 35.8 (35.6,36.0) | 35.6 (35.4,35.7) |
| 23 | 37.9 (37.7,38.1) | 37.9 (37.8,38.1) | 37.7 (37.5,37.8) |
| 24 | 39.9 (39.7,40.1) | 39.9 (39.8,40.1) | 39.7 (39.5,39.9) |
| 25 | 41.9 (41.7,42.1) | 41.9 (41.7,42.1) | 41.7 (41.5,41.9) |
| 26 | 43.8 (43.6,44.0) | 43.8 (43.7,44.0) | 43.7 (43.5,43.9) |
| 27 | 45.7 (45.5,45.9) | 45.7 (45.5,45.9) | 45.6 (45.4,45.8) |
| 28 | 47.5 (47.3,47.7) | 47.5 (47.4,47.7) | 47.4 (47.3,47.6) |
| 29 | 49.3 (49.1,49.5) | 49.3 (49.1,49.5) | 49.2 (49.0,49.4) |
| 30 | 51.0 (50.8,51.2) | 51.0 (50.8,51.2) | 50.9 (50.7,51.1) |
| 31 | 52.6 (52.4,52.8) | 52.6 (52.4,52.8) | 52.5 (52.3,52.7) |
| 32 | 54.2 (53.9,54.4) | 54.2 (54.0,54.4) | 54.0 (53.8,54.2) |
| 33 | 55.6 (55.4,55.8) | 55.7 (55.5,55.9) | 55.4 (55.2,55.6) |
| 34 | 57.0 (56.7,57.2) | 57.1 (56.9,57.4) | 56.8 (56.6,57.0) |
| 35 | 58.3 (58.0,58.5) | 58.5 (58.3,58.7) | 58.1 (57.9,58.3) |
| 36 | 59.5 (59.3,59.7) | 59.8 (59.5,60.0) | 59.5 (59.2,59.7) |
| 37 | 60.7 (60.5,61.0) | 60.9 (60.7,61.2) | 60.8 (60.5,61.0) |
| 38 | 61.9 (61.6,62.1) | 62.0 (61.7,62.2) | 61.9 (61.7,62.2) |
| 39 | 63.0 (62.7,63.3) | 62.9 (62.6,63.1) | 63.0 (62.7,63.2) |
| 40 | 64.2 (63.7,64.8) | 63.6 (63.1,64.1) | 63.7 (63.3,64.2) |

© 2025 Clayton PK et al. *JAMA Network Open*.

**eTable 6.** Means of Average Femur Length by Gestational Week in Fetuses of Women with PHDI (T1), PHDI (T2), and PHDI (T3) in the NICHD Fetal Growth Studies-Singletons, 2009-2013.

| | Mean Femur Length, mm (95% CI) | | |
|---|---|---|---|
| GA | T1 | T2 | T3 |
| 15 | 15.9 (15.7,16.1) | 16.4 (16.2,16.7) | 16.3 (16.1,16.5) |
| 16 | 19.1 (19.0,19.3) | 19.5 (19.3,19.7) | 19.3 (19.2,19.5) |
| 17 | 22.5 (22.3,22.6) | 22.7 (22.5,22.8) | 22.5 (22.3,22.6) |
| 18 | 25.8 (25.6,25.9) | 25.8 (25.7,26.0) | 25.6 (25.5,25.8) |
| 19 | 29.0 (28.8,29.1) | 28.9 (28.8,29.1) | 28.7 (28.6,28.9) |
| 20 | 32.0 (31.8,32.2) | 32.0 (31.8,32.1) | 31.7 (31.5,31.9) |
| 21 | 34.8 (34.6,35.0) | 34.8 (34.6,35.0) | 34.6 (34.4,34.8) |
| 22 | 37.5 (37.3,37.6) | 37.5 (37.4,37.7) | 37.3 (37.1,37.5) |
| 23 | 40.0 (39.8,40.1) | 40.1 (39.9,40.3) | 39.9 (39.7,40.1) |
| 24 | 42.4 (42.2,42.6) | 42.6 (42.4,42.8) | 42.4 (42.2,42.6) |
| 25 | 44.8 (44.6,45.0) | 45.0 (44.8,45.2) | 44.9 (44.7,45.1) |
| 26 | 47.3 (47.0,47.5) | 47.4 (47.2,47.6) | 47.3 (47.1,47.5) |
| 27 | 49.7 (49.5,49.9) | 49.8 (49.6,50.0) | 49.7 (49.5,49.9) |
| 28 | 52.0 (51.8,52.2) | 52.0 (51.8,52.2) | 52.0 (51.8,52.2) |
| 29 | 54.3 (54.1,54.5) | 54.3 (54.1,54.5) | 54.2 (54.0,54.4) |
| 30 | 56.5 (56.3,56.7) | 56.4 (56.2,56.6) | 56.4 (56.2,56.6) |
| 31 | 58.6 (58.3,58.8) | 58.5 (58.3,58.7) | 58.5 (58.2,58.7) |
| 32 | 60.5 (60.2,60.7) | 60.5 (60.3,60.8) | 60.4 (60.2,60.7) |
| 33 | 62.3 (62.0,62.5) | 62.5 (62.3,62.7) | 62.4 (62.1,62.6) |
| 34 | 64.0 (63.8,64.3) | 64.4 (64.1,64.6) | 64.2 (63.9,64.4) |
| 35 | 65.8 (65.5,66.0) | 66.1 (65.9,66.4) | 65.9 (65.7,66.1) |
| 36 | 67.5 (67.2,67.7) | 67.8 (67.6,68.0) | 67.5 (67.3,67.8) |
| 37 | 69.1 (68.9,69.4) | 69.3 (69.1,69.6) | 69.1 (68.8,69.3) |
| 38 | 70.6 (70.3,70.9) | 70.8 (70.5,71.0) | 70.6 (70.3,70.8) |
| 39 | 71.7 (71.3,72.0) | 72.1 (71.8,72.4) | 72.0 (71.7,72.3) |
| 40 | 72.2 (71.6,72.9) | 73.3 (72.7,73.9) | 73.4 (72.9,74.0) |

© 2025 Clayton PK et al. *JAMA Network Open*.

**eTable 7**. Means of Fractional Arm Volume by Gestational Week in Fetuses of Women with PHDI (T1), PHDI (T2), and PHDI (T3) in the NICHD Fetal Growth Studies-Singletons, 2009-2013.

| | Mean Fractional Arm Volume, cm3 (95% CI) | | |
|---|---|---|---|
| GA | T1 | T2 | T3 |
| 15 | 0.47 (0.43,0.52) | 0.50 (0.46,0.56) | 0.48 (0.44,0.51) |
| 16 | 0.72 (0.68,0.75) | 0.75 (0.71,0.78) | 0.72 (0.69,0.75) |
| 17 | 1.03 (1.01,1.06) | 1.06 (1.03,1.09) | 1.04 (1.01,1.07) |
| 18 | 1.43 (1.39,1.47) | 1.45 (1.41,1.49) | 1.43 (1.39,1.47) |
| 19 | 1.91 (1.86,1.96) | 1.92 (1.87,1.97) | 1.90 (1.85,1.95) |
| 20 | 2.47 (2.41,2.53) | 2.47 (2.42,2.53) | 2.44 (2.38,2.49) |
| 21 | 3.10 (3.03,3.17) | 3.10 (3.04,3.16) | 3.04 (2.98,3.11) |
| 22 | 3.79 (3.71,3.88) | 3.80 (3.72,3.88) | 3.73 (3.65,3.81) |
| 23 | 4.56 (4.46,4.66) | 4.57 (4.47,4.67) | 4.51 (4.41,4.60) |
| 24 | 5.38 (5.27,5.49) | 5.41 (5.31,5.52) | 5.38 (5.27,5.48) |
| 25 | 6.28 (6.16,6.40) | 6.33 (6.22,6.45) | 6.34 (6.23,6.46) |
| 26 | 7.26 (7.13,7.40) | 7.34 (7.20,7.47) | 7.41 (7.28,7.54) |
| 27 | 8.35 (8.19,8.52) | 8.44 (8.28,8.60) | 8.59 (8.43,8.75) |
| 28 | 9.59 (9.39,9.78) | 9.68 (9.49,9.86) | 9.88 (9.70,10.07) |
| 29 | 11.01 (10.81,11.22) | 11.08 (10.88,11.28) | 11.30 (11.11,11.50) |
| 30 | 12.65 (12.43,12.87) | 12.67 (12.45,12.88) | 12.86 (12.65,13.07) |
| 31 | 14.49 (14.22,14.77) | 14.45 (14.19,14.72) | 14.57 (14.32,14.82) |
| 32 | 16.53 (16.19,16.87) | 16.45 (16.12,16.78) | 16.45 (16.14,16.77) |
| 33 | 18.71 (18.32,19.11) | 18.64 (18.26,19.03) | 18.53 (18.17,18.90) |
| 34 | 21.01 (20.57,21.45) | 21.03 (20.61,21.45) | 20.82 (20.42,21.22) |
| 35 | 23.37 (22.82,23.93) | 23.54 (23.03,24.06) | 23.3 (22.84,23.77) |
| 36 | 25.76 (25.06,26.47) | 26.11 (25.45,26.78) | 25.94 (25.37,26.53) |
| 37 | 28.13 (27.33,28.95) | 28.62 (27.84,29.42) | 28.7 (28.02,29.40) |
| 38 | 30.42 (29.45,31.41) | 30.93 (30.04,31.85) | 31.49 (30.69,32.32) |
| 39 | 32.57 (30.89,34.33) | 32.89 (31.56,34.28) | 34.24 (33.09,35.43) |
| 40 | 34.52 (31.25,38.14) | 34.33 (31.86,37.00) | 36.83 (34.74,39.06) |

© 2025 Clayton PK et al. *JAMA Network Open*.

**eTable 8**. Means of Fractional Lean Arm Volume by Gestational Week in Fetuses of Women with PHDI (T1), PHDI (T2), and PHDI (T3) in the NICHD Fetal Growth Studies-Singletons, 2009-2013.

| | Mean Fractional Lean Arm Volume, $cm^3$ (95% CI) | | |
|---|---|---|---|
| GA | T1 | T2 | T3 |
| 15 | 0.21 (0.19,0.24) | 0.22 (0.20,0.25) | 0.22 (0.20,0.24) |
| 16 | 0.34 (0.32,0.36) | 0.35 (0.32,0.37) | 0.35 (0.33,0.37) |
| 17 | 0.51 (0.49,0.53) | 0.52 (0.50,0.54) | 0.52 (0.50,0.53) |
| 18 | 0.73 (0.70,0.75) | 0.73 (0.70,0.75) | 0.72 (0.70,0.75) |
| 19 | 0.98 (0.95,1.01) | 0.98 (0.95,1.01) | 0.97 (0.94,1.00) |
| 20 | 1.26 (1.22,1.30) | 1.26 (1.23,1.30) | 1.25 (1.21,1.28) |
| 21 | 1.57 (1.53,1.61) | 1.57 (1.54,1.61) | 1.55 (1.52,1.59) |
| 22 | 1.90 (1.85,1.95) | 1.91 (1.87,1.96) | 1.88 (1.84,1.93) |
| 23 | 2.25 (2.20,2.31) | 2.27 (2.21,2.32) | 2.24 (2.19,2.30) |
| 24 | 2.63 (2.57,2.70) | 2.65 (2.58,2.71) | 2.63 (2.57,2.69) |
| 25 | 3.04 (2.98,3.11) | 3.05 (2.98,3.12) | 3.05 (2.99,3.12) |
| 26 | 3.49 (3.41,3.56) | 3.48 (3.40,3.55) | 3.51 (3.44,3.58) |
| 27 | 3.97 (3.88,4.05) | 3.93 (3.85,4.02) | 4.00 (3.92,4.08) |
| 28 | 4.48 (4.38,4.59) | 4.43 (4.34,4.53) | 4.52 (4.43,4.62) |
| 29 | 5.05 (4.94,5.17) | 4.99 (4.88,5.09) | 5.09 (4.98,5.19) |
| 30 | 5.68 (5.57,5.80) | 5.60 (5.49,5.71) | 5.69 (5.58,5.80) |
| 31 | 6.38 (6.25,6.51) | 6.28 (6.15,6.41) | 6.34 (6.22,6.46) |
| 32 | 7.15 (6.99,7.32) | 7.01 (6.85,7.17) | 7.03 (6.88,7.18) |
| 33 | 8.01 (7.81,8.21) | 7.77 (7.58,7.95) | 7.77 (7.59,7.94) |
| 34 | 8.96 (8.75,9.18) | 8.54 (8.34,8.74) | 8.55 (8.37,8.74) |
| 35 | 9.97 (9.71,10.23) | 9.31 (9.09,9.54) | 9.40 (9.19,9.60) |
| 36 | 10.97 (10.64,11.31) | 10.09 (9.81,10.38) | 10.29 (10.04,10.54) |
| 37 | 11.87 (11.49,12.27) | 10.87 (10.54,11.22) | 11.23 (10.93,11.54) |
| 38 | 12.58 (12.14,13.04) | 11.66 (11.29,12.05) | 12.22 (11.88,12.58) |
| 39 | 12.99 (12.24,13.79) | 12.45 (11.92,13.01) | 13.27 (12.77,13.79) |
| 40 | 12.98 (11.55,14.59) | 13.26 (12.22,14.38) | 14.36 (13.42,15.36) |

© 2025 Clayton PK et al. *JAMA Network Open*.

**eTable 9.** Means of Fractional Fat Arm Volume by Gestational Week in Fetuses of Women with PHDI (T1), PHDI (T2), and PHDI (T3) in the NICHD Fetal Growth Studies-Singletons, 2009-2013.

| | Mean Fractional Fat Arm Volume, $cm^3$ (95% CI) | | |
|---|---|---|---|
| GA | T1 | T2 | T3 |
| 15 | 0.26 (0.22,0.30) | 0.27 (0.24,0.31) | 0.25 (0.23,0.28) |
| 16 | 0.37 (0.35,0.40) | 0.39 (0.36,0.42) | 0.37 (0.35,0.39) |
| 17 | 0.53 (0.50,0.55) | 0.53 (0.51,0.56) | 0.52 (0.50,0.54) |
| 18 | 0.71 (0.69,0.74) | 0.72 (0.69,0.74) | 0.70 (0.68,0.73) |
| 19 | 0.94 (0.91,0.98) | 0.94 (0.91,0.97) | 0.92 (0.89,0.95) |
| 20 | 1.21 (1.17,1.25) | 1.20 (1.16,1.24) | 1.18 (1.14,1.22) |
| 21 | 1.52 (1.47,1.56) | 1.51 (1.46,1.55) | 1.48 (1.44,1.52) |
| 22 | 1.86 (1.81,1.92) | 1.86 (1.81,1.91) | 1.83 (1.78,1.88) |
| 23 | 2.25 (2.19,2.32) | 2.26 (2.19,2.32) | 2.23 (2.16,2.29) |
| 24 | 2.69 (2.62,2.77) | 2.71 (2.64,2.79) | 2.69 (2.62,2.76) |
| 25 | 3.18 (3.10,3.27) | 3.23 (3.14,3.31) | 3.23 (3.15,3.31) |
| 26 | 3.73 (3.64,3.83) | 3.81 (3.72,3.90) | 3.84 (3.75,3.93) |
| 27 | 4.36 (4.25,4.47) | 4.47 (4.36,4.58) | 4.54 (4.43,4.64) |
| 28 | 5.08 (4.94,5.21) | 5.21 (5.08,5.34) | 5.31 (5.18,5.44) |
| 29 | 5.92 (5.77,6.07) | 6.05 (5.90,6.20) | 6.17 (6.02,6.31) |
| 30 | 6.90 (6.74,7.06) | 7.00 (6.85,7.16) | 7.10 (6.95,7.26) |
| 31 | 8.02 (7.84,8.22) | 8.09 (7.91,8.28) | 8.13 (7.96,8.32) |
| 32 | 9.26 (9.01,9.51) | 9.33 (9.09,9.58) | 9.28 (9.05,9.51) |
| 33 | 10.56 (10.26,10.86) | 10.73 (10.44,11.03) | 10.56 (10.29,10.85) |
| 34 | 11.86 (11.53,12.19) | 12.31 (11.99,12.64) | 12.02 (11.72,12.33) |
| 35 | 13.17 (12.78,13.57) | 14.04 (13.65,14.43) | 13.63 (13.29,13.99) |
| 36 | 14.53 (14.02,15.05) | 15.83 (15.32,16.35) | 15.37 (14.94,15.82) |
| 37 | 16.00 (15.41,16.61) | 17.57 (16.95,18.21) | 17.18 (16.65,17.72) |
| 38 | 17.67 (16.96,18.42) | 19.11 (18.41,19.85) | 18.96 (18.34,19.59) |
| 39 | 19.68 (18.37,21.09) | 20.28 (19.23,21.39) | 20.60 (19.71,21.52) |
| 40 | 22.20 (19.38,25.43) | 20.90 (18.90,23.11) | 21.98 (20.32,23.77) |

© 2025 Clayton PK et al. *JAMA Network Open*.

**eTable 10**. Means of Maximum Arm Subcutaneous Tissue Thickness (SCTT) by Gestational Week in Fetuses of Women with PHDI (T1), PHDI (T2), and PHDI (T3) in the NICHD Fetal Growth Studies-Singletons, 2009-2013.

| | Mean Maximum Arm SCTT, cm (95% CI) | | |
|---|---|---|---|
| GA | T1 | T2 | T3 |
| 15 | 0.15 (0.13,0.17) | 0.16 (0.13,0.18) | 0.14 (0.12,0.15) |
| 16 | 0.16 (0.15,0.17) | 0.16 (0.15,0.18) | 0.15 (0.15,0.16) |
| 17 | 0.18 (0.17,0.18) | 0.17 (0.17,0.18) | 0.17 (0.17,0.18) |
| 18 | 0.19 (0.19,0.20) | 0.19 (0.18,0.20) | 0.19 (0.19,0.20) |
| 19 | 0.21 (0.20,0.22) | 0.21 (0.20,0.21) | 0.21 (0.21,0.22) |
| 20 | 0.23 (0.23,0.24) | 0.23 (0.22,0.24) | 0.23 (0.23,0.24) |
| 21 | 0.26 (0.25,0.26) | 0.25 (0.24,0.26) | 0.25 (0.25,0.26) |
| 22 | 0.28 (0.27,0.29) | 0.28 (0.27,0.28) | 0.27 (0.27,0.28) |
| 23 | 0.30 (0.29,0.31) | 0.30 (0.29,0.31) | 0.30 (0.29,0.31) |
| 24 | 0.32 (0.31,0.33) | 0.32 (0.32,0.33) | 0.32 (0.31,0.33) |
| 25 | 0.34 (0.33,0.35) | 0.35 (0.34,0.36) | 0.34 (0.33,0.35) |
| 26 | 0.36 (0.35,0.37) | 0.37 (0.36,0.38) | 0.36 (0.35,0.37) |
| 27 | 0.38 (0.37,0.39) | 0.40 (0.38,0.41) | 0.39 (0.38,0.40) |
| 28 | 0.40 (0.39,0.41) | 0.42 (0.41,0.43) | 0.41 (0.40,0.43) |
| 29 | 0.43 (0.41,0.44) | 0.45 (0.43,0.46) | 0.44 (0.43,0.45) |
| 30 | 0.46 (0.45,0.47) | 0.47 (0.46,0.49) | 0.47 (0.46,0.48) |
| 31 | 0.49 (0.48,0.51) | 0.50 (0.49,0.52) | 0.50 (0.49,0.51) |
| 32 | 0.53 (0.51,0.54) | 0.53 (0.52,0.55) | 0.53 (0.52,0.55) |
| 33 | 0.56 (0.54,0.58) | 0.56 (0.55,0.58) | 0.56 (0.54,0.58) |
| 34 | 0.60 (0.58,0.62) | 0.59 (0.57,0.61) | 0.59 (0.57,0.61) |
| 35 | 0.63 (0.61,0.65) | 0.62 (0.60,0.64) | 0.62 (0.60,0.64) |
| 36 | 0.65 (0.63,0.68) | 0.65 (0.62,0.67) | 0.65 (0.63,0.67) |
| 37 | 0.67 (0.65,0.70) | 0.67 (0.64,0.70) | 0.68 (0.66,0.70) |
| 38 | 0.68 (0.65,0.72) | 0.69 (0.66,0.72) | 0.71 (0.68,0.74) |
| 39 | 0.69 (0.64,0.74) | 0.71 (0.67,0.75) | 0.74 (0.71,0.78) |
| 40 | 0.67 (0.58,0.78) | 0.72 (0.65,0.80) | 0.78 (0.71,0.85) |

© 2025 Clayton PK et al. *JAMA Network Open*.

**eTable 11.** Means of Abdominal Area by Gestational Week in Fetuses of Women with PHDI (T1), PHDI (T2), and PHDI (T3) in the NICHD Fetal Growth Studies-Singletons, 2009-2013.

| | Mean Abdominal Area, mm$^2$ (95% CI) | | |
|---|---|---|---|
| GA | T1 | T2 | T3 |
| 15 | 655.7 (634.7,677.5) | 697.3 (672.3,723.3) | 695.6 (675.2,716.7) |
| 16 | 849.1 (833.1,865.5) | 881.5 (862.7,900.7) | 873.3 (857.9,888.9) |
| 17 | 1067.6 (1053.9,1081.4) | 1090.0 (1075.0,1105.1) | 1075.9 (1062.6,1089.3) |
| 18 | 1307.2 (1291.5,1323.1) | 1320.9 (1305.3,1336.7) | 1302.6 (1287.2,1318.2) |
| 19 | 1563.9 (1544.7,1583.4) | 1572.1 (1553.5,1590.9) | 1552.0 (1533.3,1570.9) |
| 20 | 1833.8 (1811.9,1855.9) | 1841.1 (1820.0,1862.3) | 1822.1 (1801.0,1843.5) |
| 21 | 2114.1 (2090.8,2137.7) | 2125.7 (2103.3,2148.4) | 2110.9 (2088.3,2133.7) |
| 22 | 2403.8 (2378.8,2429.1) | 2424.8 (2400.6,2449.2) | 2416.2 (2391.8,2440.8) |
| 23 | 2704.2 (2675.8,2733.0) | 2737.8 (2710.2,2765.7) | 2736.3 (2708.6,2764.3) |
| 24 | 3019.4 (2986.5,3052.7) | 3066.0 (3033.9,3098.4) | 3070.1 (3038.2,3102.4) |
| 25 | 3354.9 (3318.8,3391.5) | 3411.4 (3376.1,3447.0) | 3417.1 (3382.1,3452.5) |
| 26 | 3711.8 (3673.8,3750.2) | 3774.3 (3737.5,3811.4) | 3777.3 (3740.6,3814.3) |
| 27 | 4089.5 (4049.8,4129.5) | 4154.5 (4116.2,4193.1) | 4151.1 (4112.8,4189.8) |
| 28 | 4487.1 (4444.1,4530.4) | 4552.2 (4510.9,4594.0) | 4540.5 (4498.9,4582.5) |
| 29 | 4903.7 (4855.8,4952.0) | 4968.2 (4921.9,5014.9) | 4948.3 (4901.9,4995.2) |
| 30 | 5338.0 (5285.3,5391.1) | 5403.5 (5352.5,5455.1) | 5379.0 (5327.8,5430.6) |
| 31 | 5788.4 (5732.9,5844.5) | 5859.7 (5805.9,5914.1) | 5837.4 (5783.3,5892.0) |
| 32 | 6252.3 (6193.0,6312.2) | 6334.1 (6276.7,6391.9) | 6319.6 (6261.9,6377.8) |
| 33 | 6725.8 (6659.1,6793.2) | 6820.0 (6755.3,6885.4) | 6812.9 (6748.0,6878.4) |
| 34 | 7204.3 (7128.9,7280.4) | 7309.3 (7235.8,7383.6) | 7300.9 (7227.5,7375.0) |
| 35 | 7682.5 (7602.3,7763.6) | 7792.1 (7713.6,7871.3) | 7763.2 (7685.0,7842.2) |
| 36 | 8155.1 (8070.8,8240.3) | 8260.3 (8178.3,8343.2) | 8184.0 (8102.7,8266.1) |
| 37 | 8618.1 (8521.0,8716.3) | 8726.8 (8632.0,8822.5) | 8590.3 (8498.3,8683.3) |
| 38 | 9068.2 (8960.0,9177.8) | 9215.4 (9109.0,9323.0) | 9031.8 (8929.1,9135.6) |
| 39 | 9502.6 (9369.4,9637.7) | 9756.0 (9631.9,9881.7) | 9569.1 (9449.0,9690.6) |
| 40 | 9918.5 (9647.3,10197.3) | 10385.6 (10141.1,10636.0) | 10277.9 (10047.9,10513.2) |

© 2025 Clayton PK et al. *JAMA Network Open*.

**eTable 12.** Means of Maximum Abdominal Subcutaneous Tissue Thickness (SCTT) by Gestational Week in Fetuses of Women with PHDI (T1), PHDI (T2), and PHDI (T3) in the NICHD Fetal Growth Studies-Singletons, 2009-2013.

| | Mean Maximum Abdominal SCTT, cm (95% CI) | | |
|---|---|---|---|
| GA | T1 | T2 | T3 |
| 15 | 1.38 (1.25,1.51) | 1.36 (1.23,1.51) | 1.38 (1.26,1.50) |
| 16 | 1.52 (1.44,1.60) | 1.53 (1.44,1.63) | 1.51 (1.44,1.59) |
| 17 | 1.64 (1.59,1.70) | 1.69 (1.62,1.75) | 1.64 (1.58,1.69) |
| 18 | 1.76 (1.71,1.82) | 1.83 (1.77,1.89) | 1.76 (1.70,1.82) |
| 19 | 1.87 (1.81,1.93) | 1.96 (1.89,2.02) | 1.87 (1.81,1.94) |
| 20 | 1.97 (1.91,2.03) | 2.07 (2.00,2.13) | 1.98 (1.92,2.05) |
| 21 | 2.06 (2.00,2.13) | 2.17 (2.11,2.23) | 2.09 (2.03,2.15) |
| 22 | 2.15 (2.10,2.22) | 2.27 (2.21,2.33) | 2.19 (2.13,2.25) |
| 23 | 2.25 (2.18,2.31) | 2.36 (2.30,2.43) | 2.29 (2.23,2.36) |
| 24 | 2.35 (2.28,2.42) | 2.46 (2.39,2.53) | 2.40 (2.33,2.47) |
| 25 | 2.45 (2.38,2.53) | 2.58 (2.51,2.65) | 2.51 (2.44,2.58) |
| 26 | 2.58 (2.50,2.65) | 2.71 (2.64,2.78) | 2.63 (2.56,2.70) |
| 27 | 2.71 (2.64,2.78) | 2.85 (2.78,2.92) | 2.75 (2.68,2.82) |
| 28 | 2.85 (2.78,2.93) | 3.00 (2.93,3.08) | 2.88 (2.81,2.96) |
| 29 | 3.01 (2.93,3.09) | 3.17 (3.09,3.25) | 3.03 (2.95,3.10) |
| 30 | 3.18 (3.09,3.26) | 3.34 (3.25,3.43) | 3.18 (3.09,3.26) |
| 31 | 3.36 (3.27,3.45) | 3.52 (3.43,3.61) | 3.34 (3.25,3.42) |
| 32 | 3.54 (3.45,3.64) | 3.70 (3.61,3.79) | 3.50 (3.42,3.59) |
| 33 | 3.73 (3.63,3.83) | 3.88 (3.78,3.99) | 3.68 (3.58,3.78) |
| 34 | 3.91 (3.80,4.03) | 4.07 (3.95,4.18) | 3.85 (3.74,3.96) |
| 35 | 4.07 (3.95,4.19) | 4.25 (4.13,4.38) | 4.01 (3.90,4.13) |
| 36 | 4.21 (4.09,4.34) | 4.44 (4.31,4.57) | 4.17 (4.05,4.29) |
| 37 | 4.34 (4.20,4.48) | 4.62 (4.47,4.76) | 4.31 (4.18,4.45) |
| 38 | 4.45 (4.30,4.61) | 4.77 (4.61,4.93) | 4.46 (4.31,4.61) |
| 39 | 4.58 (4.39,4.77) | 4.89 (4.71,5.07) | 4.60 (4.44,4.77) |
| 40 | 4.73 (4.35,5.13) | 4.95 (4.61,5.32) | 4.76 (4.45,5.08) |

© 2025 Clayton PK et al. *JAMA Network Open*.

**eTable 13.** Means of Fractional Thigh Volume by Gestational Week in Fetuses of Women with PHDI (T1), PHDI (T2), and PHDI (T3) in the NICHD Fetal Growth Studies-Singletons, 2009-2013.

| | Mean Fractional Thigh Volume, $cm^3$ (95% CI) | | |
|---|---|---|---|
| GA | T1 | T2 | T3 |
| 15 | 0.68 (0.62,0.75) | 0.65 (0.59,0.72) | 0.70 (0.65,0.76) |
| 16 | 1.11 (1.06,1.16) | 1.07 (1.02,1.13) | 1.10 (1.06,1.15) |
| 17 | 1.69 (1.65,1.74) | 1.66 (1.62,1.71) | 1.66 (1.62,1.71) |
| 18 | 2.45 (2.38,2.53) | 2.44 (2.38,2.51) | 2.40 (2.33,2.46) |
| 19 | 3.40 (3.30,3.49) | 3.42 (3.33,3.51) | 3.34 (3.25,3.43) |
| 20 | 4.52 (4.41,4.64) | 4.58 (4.48,4.69) | 4.49 (4.39,4.60) |
| 21 | 5.83 (5.70,5.97) | 5.92 (5.80,6.05) | 5.86 (5.73,5.98) |
| 22 | 7.33 (7.17,7.50) | 7.44 (7.28,7.61) | 7.43 (7.27,7.59) |
| 23 | 9.04 (8.84,9.25) | 9.14 (8.94,9.34) | 9.19 (8.99,9.39) |
| 24 | 10.97 (10.74,11.20) | 11.00 (10.78,11.23) | 11.13 (10.91,11.35) |
| 25 | 13.12 (12.86,13.37) | 13.05 (12.81,13.31) | 13.25 (13.01,13.49) |
| 26 | 15.49 (15.19,15.80) | 15.32 (15.03,15.61) | 15.57 (15.29,15.86) |
| 27 | 18.11 (17.73,18.49) | 17.84 (17.48,18.20) | 18.14 (17.79,18.49) |
| 28 | 20.99 (20.56,21.43) | 20.71 (20.29,21.13) | 21.02 (20.61,21.43) |
| 29 | 24.18 (23.72,24.64) | 24.02 (23.58,24.47) | 24.31 (23.88,24.75) |
| 30 | 27.72 (27.23,28.22) | 27.84 (27.35,28.34) | 28.06 (27.59,28.54) |
| 31 | 31.69 (31.08,32.32) | 32.17 (31.56,32.80) | 32.27 (31.69,32.86) |
| 32 | 36.19 (35.42,36.97) | 37.01 (36.23,37.81) | 36.93 (36.20,37.67) |
| 33 | 41.32 (40.45,42.22) | 42.32 (41.43,43.22) | 42.01 (41.17,42.86) |
| 34 | 47.10 (46.09,48.14) | 47.99 (46.99,49.00) | 47.47 (46.54,48.42) |
| 35 | 53.40 (52.09,54.73) | 53.87 (52.64,55.12) | 53.32 (52.20,54.46) |
| 36 | 59.98 (58.32,61.68) | 59.73 (58.18,61.33) | 59.51 (58.12,60.94) |
| 37 | 66.49 (64.56,68.48) | 65.31 (63.50,67.17) | 66.03 (64.37,67.73) |
| 38 | 72.50 (70.10,74.98) | 70.25 (68.18,72.38) | 72.83 (70.87,74.85) |
| 39 | 77.44 (73.3,81.82) | 74.19 (71.11,77.41) | 79.88 (76.96,82.91) |
| 40 | 80.74 (72.98,89.33) | 76.79 (71.17,82.86) | 87.12 (81.74,92.85) |

© 2025 Clayton PK et al. *JAMA Network Open*.

**eTable 14.** Means of Fractional Lean Thigh Volume by Gestational Week in Fetuses of Women with PHDI (T1), PHDI (T2), and PHDI (T3) in the NICHD Fetal Growth Studies-Singletons, 2009-2013.

| | Mean Fractional Lean Thigh Volume, $cm^3$ (95% CI) | | |
|---|---|---|---|
| GA | T1 | T2 | T3 |
| 15 | 0.33 (0.29,0.37) | 0.33 (0.29,0.37) | 0.34 (0.31,0.37) |
| 16 | 0.55 (0.52,0.58) | 0.56 (0.52,0.60) | 0.55 (0.53,0.58) |
| 17 | 0.87 (0.83,0.90) | 0.89 (0.85,0.92) | 0.87 (0.84,0.90) |
| 18 | 1.29 (1.25,1.34) | 1.33 (1.29,1.37) | 1.29 (1.25,1.33) |
| 19 | 1.83 (1.77,1.90) | 1.90 (1.84,1.96) | 1.84 (1.78,1.90) |
| 20 | 2.49 (2.41,2.57) | 2.59 (2.51,2.66) | 2.52 (2.44,2.59) |
| 21 | 3.26 (3.17,3.35) | 3.40 (3.31,3.48) | 3.33 (3.24,3.42) |
| 22 | 4.14 (4.03,4.25) | 4.30 (4.20,4.41) | 4.26 (4.16,4.37) |
| 23 | 5.12 (4.98,5.26) | 5.31 (5.17,5.45) | 5.30 (5.16,5.44) |
| 24 | 6.23 (6.06,6.39) | 6.39 (6.22,6.56) | 6.42 (6.26,6.58) |
| 25 | 7.44 (7.27,7.63) | 7.55 (7.37,7.74) | 7.62 (7.45,7.79) |
| 26 | 8.77 (8.57,8.97) | 8.80 (8.60,9.00) | 8.89 (8.70,9.08) |
| 27 | 10.20 (9.96,10.44) | 10.14 (9.91,10.37) | 10.25 (10.02,10.47) |
| 28 | 11.73 (11.44,12.02) | 11.61 (11.34,11.89) | 11.72 (11.46,12.00) |
| 29 | 13.36 (13.05,13.67) | 13.26 (12.96,13.56) | 13.37 (13.09,13.67) |
| 30 | 15.11 (14.79,15.43) | 15.11 (14.80,15.43) | 15.23 (14.93,15.54) |
| 31 | 17.01 (16.64,17.39) | 17.16 (16.80,17.54) | 17.28 (16.93,17.64) |
| 32 | 19.13 (18.66,19.61) | 19.39 (18.93,19.87) | 19.53 (19.09,19.97) |
| 33 | 21.54 (20.99,22.10) | 21.76 (21.21,22.31) | 21.93 (21.42,22.46) |
| 34 | 24.31 (23.71,24.93) | 24.21 (23.62,24.80) | 24.47 (23.91,25.03) |
| 35 | 27.38 (26.65,28.14) | 26.69 (26.02,27.38) | 27.12 (26.49,27.76) |
| 36 | 30.60 (29.65,31.59) | 29.16 (28.32,30.03) | 29.86 (29.08,30.66) |
| 37 | 33.75 (32.62,34.91) | 31.56 (30.57,32.57) | 32.69 (31.76,33.64) |
| 38 | 36.51 (35.19,37.89) | 33.81 (32.71,34.94) | 35.58 (34.51,36.68) |
| 39 | 38.55 (36.30,40.94) | 35.85 (34.26,37.51) | 38.52 (36.96,40.14) |
| 40 | 39.50 (35.15,44.39) | 37.60 (34.55,40.93) | 41.49 (38.51,44.70) |

© 2025 Clayton PK et al. *JAMA Network Open*.

**eTable 15.** Means of Fractional Fat Thigh Volume by Gestational Week in Fetuses of Women with PHDI (T1), PHDI (T2), and PHDI (T3) in the NICHD Fetal Growth Studies-Singletons, 2009-2013.

| | Mean Fractional Fat Thigh Volume, $cm^3$ (95% CI) | | |
|---|---|---|---|
| GA | T1 | T2 | T3 |
| 15 | 0.34 (0.30,0.38) | 0.34 (0.30,0.39) | 0.34 (0.31,0.38) |
| 16 | 0.54 (0.51,0.58) | 0.53 (0.50,0.57) | 0.54 (0.51,0.57) |
| 17 | 0.82 (0.79,0.85) | 0.79 (0.76,0.82) | 0.79 (0.76,0.82) |
| 18 | 1.16 (1.12,1.21) | 1.11 (1.07,1.15) | 1.12 (1.08,1.16) |
| 19 | 1.57 (1.51,1.63) | 1.50 (1.45,1.55) | 1.51 (1.46,1.56) |
| 20 | 2.04 (1.97,2.11) | 1.96 (1.91,2.02) | 1.98 (1.92,2.04) |
| 21 | 2.56 (2.49,2.64) | 2.49 (2.43,2.56) | 2.51 (2.45,2.58) |
| 22 | 3.15 (3.06,3.24) | 3.09 (3.01,3.18) | 3.12 (3.04,3.21) |
| 23 | 3.83 (3.71,3.94) | 3.78 (3.67,3.88) | 3.82 (3.72,3.93) |
| 24 | 4.61 (4.49,4.74) | 4.55 (4.43,4.68) | 4.62 (4.50,4.74) |
| 25 | 5.53 (5.39,5.67) | 5.44 (5.31,5.58) | 5.53 (5.40,5.66) |
| 26 | 6.58 (6.42,6.75) | 6.46 (6.31,6.62) | 6.58 (6.43,6.73) |
| 27 | 7.79 (7.59,8.00) | 7.64 (7.45,7.84) | 7.78 (7.59,7.97) |
| 28 | 9.17 (8.92,9.42) | 9.02 (8.78,9.26) | 9.17 (8.94,9.40) |
| 29 | 10.73 (10.46,11.00) | 10.64 (10.38,10.90) | 10.79 (10.53,11.05) |
| 30 | 12.49 (12.2,12.78) | 12.54 (12.26,12.84) | 12.66 (12.38,12.95) |
| 31 | 14.48 (14.12,14.85) | 14.77 (14.40,15.14) | 14.80 (14.46,15.16) |
| 32 | 16.77 (16.30,17.25) | 17.31 (16.83,17.81) | 17.21 (16.76,17.67) |
| 33 | 19.41 (18.84,19.98) | 20.18 (19.61,20.78) | 19.86 (19.32,20.41) |
| 34 | 22.43 (21.78,23.09) | 23.34 (22.69,24.01) | 22.73 (22.13,23.34) |
| 35 | 25.77 (24.94,26.62) | 26.68 (25.88,27.51) | 25.82 (25.11,26.55) |
| 36 | 29.28 (28.19,30.40) | 30.05 (29.02,31.13) | 29.14 (28.23,30.06) |
| 37 | 32.74 (31.45,34.07) | 33.22 (31.99,34.51) | 32.70 (31.60,33.83) |
| 38 | 35.84 (34.28,37.47) | 35.93 (34.53,37.39) | 36.53 (35.23,37.88) |
| 39 | 38.23 (35.50,41.16) | 37.88 (35.80,40.09) | 40.67 (38.71,42.73) |
| 40 | 39.52 (34.32,45.51) | 38.80 (34.93,43.10) | 45.16 (41.38,49.29) |

© 2025 Clayton PK et al. *JAMA Network Open*.

**eTable 16.** Means of Maximum Thigh Subcutaneous Tissue Thickness (SCTT) by Gestational Week in Fetuses of Women with PHDI (T1), PHDI (T2), and PHDI (T3) in the NICHD Fetal Growth Studies-Singletons, 2009-2013.

| | Mean Maximum Thigh SCTT, cm (95% CI) | | |
|---|---|---|---|
| GA | T1 | T2 | T3 |
| 15 | 0.16 (0.14,0.18) | 0.16 (0.14,0.19) | 0.16 (0.15,0.18) |
| 16 | 0.19 (0.17,0.20) | 0.18 (0.17,0.20) | 0.18 (0.17,0.20) |
| 17 | 0.21 (0.20,0.22) | 0.21 (0.20,0.22) | 0.21 (0.20,0.22) |
| 18 | 0.24 (0.23,0.25) | 0.23 (0.22,0.24) | 0.23 (0.22,0.24) |
| 19 | 0.26 (0.25,0.27) | 0.25 (0.24,0.26) | 0.25 (0.24,0.26) |
| 20 | 0.28 (0.27,0.29) | 0.27 (0.27,0.28) | 0.28 (0.27,0.29) |
| 21 | 0.30 (0.29,0.31) | 0.30 (0.29,0.31) | 0.30 (0.29,0.31) |
| 22 | 0.32 (0.31,0.33) | 0.32 (0.31,0.33) | 0.32 (0.31,0.33) |
| 23 | 0.34 (0.33,0.35) | 0.35 (0.34,0.36) | 0.35 (0.34,0.36) |
| 24 | 0.36 (0.35,0.37) | 0.37 (0.36,0.38) | 0.37 (0.36,0.38) |
| 25 | 0.38 (0.37,0.40) | 0.40 (0.39,0.41) | 0.39 (0.38,0.40) |
| 26 | 0.41 (0.40,0.42) | 0.43 (0.41,0.44) | 0.42 (0.41,0.43) |
| 27 | 0.44 (0.43,0.46) | 0.45 (0.44,0.47) | 0.44 (0.43,0.46) |
| 28 | 0.47 (0.46,0.49) | 0.49 (0.47,0.50) | 0.47 (0.46,0.49) |
| 29 | 0.51 (0.49,0.52) | 0.52 (0.51,0.54) | 0.50 (0.49,0.52) |
| 30 | 0.55 (0.53,0.56) | 0.56 (0.54,0.57) | 0.54 (0.52,0.55) |
| 31 | 0.58 (0.57,0.60) | 0.60 (0.58,0.61) | 0.58 (0.56,0.59) |
| 32 | 0.62 (0.60,0.64) | 0.64 (0.62,0.66) | 0.62 (0.60,0.64) |
| 33 | 0.66 (0.64,0.68) | 0.68 (0.66,0.70) | 0.67 (0.64,0.69) |
| 34 | 0.69 (0.67,0.72) | 0.72 (0.69,0.74) | 0.71 (0.69,0.74) |
| 35 | 0.73 (0.70,0.76) | 0.76 (0.73,0.78) | 0.76 (0.74,0.79) |
| 36 | 0.76 (0.73,0.80) | 0.79 (0.76,0.83) | 0.81 (0.78,0.84) |
| 37 | 0.81 (0.77,0.85) | 0.83 (0.79,0.87) | 0.85 (0.81,0.88) |
| 38 | 0.86 (0.82,0.90) | 0.86 (0.82,0.90) | 0.88 (0.84,0.92) |
| 39 | 0.93 (0.86,1.00) | 0.89 (0.84,0.94) | 0.91 (0.86,0.96) |
| 40 | 1.02 (0.88,1.18) | 0.91 (0.82,1.02) | 0.92 (0.83,1.01) |

© 2025 Clayton PK et al. *JAMA Network Open*.

**eTable 17.** Means of Cerebellar Volume by Gestational Week in Fetuses of Women with PHDI (T1), PHDI (T2), and PHDI (T3) in the NICHD Fetal Growth Studies-Singletons, 2009-2013.

| | Mean Cerebellar Volume, $cm^3$ (95% CI) | | |
|---|---|---|---|
| GA | T1 | T2 | T3 |
| 15 | 0.42 (0.36,0.50) | 0.50 (0.42,0.59) | 0.39 (0.34,0.45) |
| 16 | 0.57 (0.52,0.62) | 0.63 (0.58,0.69) | 0.55 (0.51,0.59) |
| 17 | 0.75 (0.71,0.78) | 0.80 (0.76,0.84) | 0.74 (0.71,0.78) |
| 18 | 0.96 (0.92,1.01) | 1.00 (0.95,1.04) | 0.96 (0.92,1.01) |
| 19 | 1.22 (1.17,1.27) | 1.24 (1.19,1.29) | 1.21 (1.16,1.27) |
| 20 | 1.52 (1.46,1.58) | 1.53 (1.47,1.59) | 1.50 (1.44,1.56) |
| 21 | 1.88 (1.81,1.95) | 1.87 (1.81,1.94) | 1.83 (1.77,1.90) |
| 22 | 2.30 (2.21,2.39) | 2.28 (2.20,2.37) | 2.23 (2.14,2.31) |
| 23 | 2.80 (2.70,2.91) | 2.76 (2.66,2.87) | 2.70 (2.61,2.80) |
| 24 | 3.40 (3.28,3.52) | 3.33 (3.22,3.45) | 3.28 (3.17,3.39) |
| 25 | 4.09 (3.96,4.23) | 3.99 (3.86,4.12) | 3.96 (3.84,4.08) |
| 26 | 4.89 (4.73,5.05) | 4.76 (4.60,4.92) | 4.75 (4.61,4.90) |
| 27 | 5.79 (5.59,5.99) | 5.65 (5.46,5.85) | 5.66 (5.47,5.85) |
| 28 | 6.79 (6.56,7.02) | 6.69 (6.47,6.91) | 6.66 (6.45,6.88) |
| 29 | 7.87 (7.64,8.11) | 7.87 (7.64,8.11) | 7.74 (7.52,7.98) |
| 30 | 9.04 (8.77,9.31) | 9.18 (8.91,9.46) | 8.90 (8.65,9.17) |
| 31 | 10.26 (9.93,10.60) | 10.56 (10.23,10.91) | 10.13 (9.82,10.44) |
| 32 | 11.53 (11.16,11.92) | 11.96 (11.58,12.36) | 11.42 (11.07,11.77) |
| 33 | 12.84 (12.42,13.27) | 13.34 (12.91,13.78) | 12.75 (12.37,13.15) |
| 34 | 14.18 (13.67,14.72) | 14.68 (14.17,15.2) | 14.14 (13.69,14.59) |
| 35 | 15.56 (14.90,16.24) | 15.97 (15.34,16.63) | 15.56 (15.01,16.13) |
| 36 | 16.97 (16.17,17.81) | 17.24 (16.47,18.04) | 17.02 (16.35,17.71) |
| 37 | 18.44 (17.46,19.48) | 18.48 (17.54,19.48) | 18.51 (17.72,19.34) |
| 38 | 19.99 (18.54,21.54) | 19.74 (18.40,21.18) | 20.04 (19.03,21.10) |
| 39 | 21.62 (19.13,24.44) | 21.04 (18.83,23.52) | 21.61 (20.08,23.26) |
| 40 | 23.40 (19.10,28.66) | 22.44 (18.73,26.89) | 23.24 (20.67,26.13) |

© 2025 Clayton PK et al. *JAMA Network Open*.

**eTable 18.** Means of Average Lung Volume by Gestational Week in Fetuses of Women with PHDI (T1), PHDI (T2), and PHDI (T3) in the NICHD Fetal Growth Studies-Singletons, 2009-2013.

| | Mean Lung Volume, mm$^2$ (95% CI) | | |
|---|---|---|---|
| GA | T1 | T2 | T3 |
| 15 | 1.69 (0.98,2.93) | 1.15 (0.61,2.16) | 1.20 (0.79,1.81) |
| 16 | 1.80 (1.48,2.19) | 1.87 (1.56,2.24) | 1.61 (1.39,1.86) |
| 17 | 2.13 (1.81,2.52) | 2.57 (2.11,3.13) | 2.12 (1.83,2.45) |
| 18 | 2.70 (2.34,3.12) | 3.18 (2.65,3.83) | 2.73 (2.38,3.13) |
| 19 | 3.51 (3.05,4.03) | 3.77 (3.27,4.35) | 3.46 (3.00,4.00) |
| 20 | 4.49 (3.80,5.29) | 4.53 (3.89,5.26) | 4.34 (3.67,5.13) |
| 21 | 5.58 (4.75,6.56) | 5.58 (4.81,6.47) | 5.37 (4.58,6.31) |
| 22 | 6.77 (5.92,7.75) | 6.97 (6.14,7.91) | 6.58 (5.76,7.51) |
| 23 | 8.06 (7.20,9.01) | 8.71 (7.84,9.69) | 7.98 (7.17,8.87) |
| 24 | 9.43 (8.42,10.55) | 10.76 (9.69,11.94) | 9.58 (8.67,10.58) |
| 25 | 10.89 (9.68,12.26) | 12.96 (11.63,14.44) | 11.4 (10.29,12.63) |
| 26 | 12.47 (11.1,14.01) | 15.24 (13.67,16.98) | 13.44 (12.12,14.90) |
| 27 | 14.17 (12.65,15.87) | 17.53 (15.72,19.55) | 15.65 (14.10,17.37) |
| 28 | 16.04 (14.31,17.97) | 19.80 (17.69,22.16) | 18.00 (16.15,20.06) |
| 29 | 18.13 (16.14,20.37) | 22.02 (19.58,24.77) | 20.40 (18.18,22.88) |
| 30 | 20.53 (18.28,23.05) | 24.19 (21.48,27.24) | 22.75 (20.21,25.60) |
| 31 | 23.28 (20.75,26.11) | 26.30 (23.38,29.59) | 24.99 (22.21,28.11) |
| 32 | 26.31 (23.19,29.85) | 28.32 (24.95,32.14) | 27.16 (24.07,30.64) |
| 33 | 29.48 (25.38,34.25) | 30.20 (26.08,34.98) | 29.35 (25.76,33.43) |
| 34 | 32.58 (27.41,38.73) | 31.91 (26.96,37.77) | 31.70 (27.48,36.57) |
| 35 | 35.31 (29.34,42.49) | 33.42 (27.90,40.03) | 34.41 (29.56,40.06) |
| 36 | 37.34 (30.90,45.11) | 34.69 (29.07,41.40) | 37.71 (32.25,44.10) |
| 37 | 38.30 (30.79,47.65) | 35.70 (30.15,42.28) | 41.95 (35.41,49.70) |
| 38 | 37.93 (27.28,52.73) | 36.44 (29.59,44.89) | 47.60 (38.06,59.55) |
| 39 | 36.05 (20.88,62.22) | 36.90 (26.11,52.16) | 55.40 (39.20,78.29) |
| 40 | 32.71 (13.72,77.96) | 37.08 (20.72,66.36) | 66.44 (38.66,114.17) |

© 2025 Clayton PK et al. *JAMA Network Open*.

**eTable 19.** Means of Kidney by Gestational Week in Fetuses of Women with PHDI (T1), PHDI (T2), and PHDI (T3) in the NICHD Fetal Growth Studies-Singletons, 2009-2013.

| | Mean Kidney Volume, mm$^2$ (95% CI) | | |
|---|---|---|---|
| GA | T1 | T2 | T3 |
| 15 | 0.21 (0.16,0.28) | 0.41 (0.30,0.57) | 0.31 (0.23,0.40) |
| 16 | 0.36 (0.31,0.42) | 0.51 (0.43,0.61) | 0.41 (0.35,0.47) |
| 17 | 0.56 (0.51,0.61) | 0.64 (0.58,0.71) | 0.54 (0.50,0.59) |
| 18 | 0.78 (0.72,0.85) | 0.81 (0.75,0.88) | 0.72 (0.66,0.78) |
| 19 | 1.03 (0.95,1.13) | 1.03 (0.95,1.12) | 0.94 (0.86,1.02) |
| 20 | 1.29 (1.19,1.40) | 1.31 (1.22,1.41) | 1.20 (1.11,1.31) |
| 21 | 1.55 (1.45,1.67) | 1.65 (1.54,1.76) | 1.53 (1.42,1.64) |
| 22 | 1.84 (1.72,1.96) | 2.05 (1.92,2.18) | 1.90 (1.78,2.03) |
| 23 | 2.16 (2.01,2.32) | 2.50 (2.34,2.68) | 2.32 (2.18,2.48) |
| 24 | 2.57 (2.40,2.75) | 2.99 (2.79,3.20) | 2.78 (2.61,2.96) |
| 25 | 3.07 (2.88,3.28) | 3.50 (3.29,3.72) | 3.27 (3.08,3.47) |
| 26 | 3.68 (3.47,3.91) | 4.03 (3.81,4.27) | 3.79 (3.59,4.01) |
| 27 | 4.38 (4.12,4.65) | 4.60 (4.34,4.87) | 4.35 (4.11,4.59) |
| 28 | 5.14 (4.83,5.48) | 5.20 (4.90,5.51) | 4.94 (4.67,5.23) |
| 29 | 5.93 (5.57,6.30) | 5.85 (5.51,6.20) | 5.60 (5.29,5.93) |
| 30 | 6.67 (6.30,7.05) | 6.57 (6.22,6.94) | 6.32 (6.00,6.67) |
| 31 | 7.35 (6.96,7.76) | 7.34 (6.96,7.74) | 7.11 (6.76,7.47) |
| 32 | 7.99 (7.53,8.48) | 8.11 (7.65,8.60) | 7.91 (7.50,8.35) |
| 33 | 8.62 (8.08,9.20) | 8.84 (8.29,9.43) | 8.70 (8.21,9.22) |
| 34 | 9.27 (8.71,9.88) | 9.44 (8.86,10.06) | 9.40 (8.88,9.96) |
| 35 | 9.99 (9.40,10.62) | 9.92 (9.33,10.54) | 10.00 (9.46,10.57) |
| 36 | 10.76 (10.03,11.55) | 10.38 (9.71,11.09) | 10.55 (9.93,11.21) |
| 37 | 11.59 (10.70,12.55) | 10.99 (10.21,11.82) | 11.13 (10.41,11.90) |
| 38 | 12.46 (11.43,13.59) | 11.94 (11.07,12.88) | 11.84 (11.06,12.67) |
| 39 | 13.36 (11.68,15.28) | 13.53 (12.16,15.05) | 12.81 (11.74,13.98) |
| 40 | 14.27 (10.89,18.71) | 16.20 (13.07,20.08) | 14.23 (12.00,16.88) |

© 2025 Clayton PK et al. *JAMA Network Open*.

**eTable 20.** Means of Liver by Gestational Week in Fetuses of Women with PHDI (T1), PHDI (T2), and PHDI (T3) in the NICHD Fetal Growth Studies-Singletons, 2009-2013.

| | Mean Liver Volume, $mm^2$ (95% CI) | | |
|---|---|---|---|
| GA | T1 | T2 | T3 |
| 15 | 2.90 (2.38,3.54) | 2.69 (2.14,3.37) | 2.72 (2.29,3.22) |
| 16 | 3.86 (3.49,4.27) | 3.88 (3.45,4.36) | 3.80 (3.47,4.16) |
| 17 | 5.09 (4.77,5.42) | 5.37 (5.01,5.75) | 5.20 (4.88,5.54) |
| 18 | 6.64 (6.23,7.08) | 7.17 (6.73,7.63) | 6.95 (6.52,7.41) |
| 19 | 8.58 (8.05,9.14) | 9.28 (8.74,9.86) | 9.10 (8.55,9.70) |
| 20 | 10.95 (10.35,11.59) | 11.72 (11.11,12.36) | 11.70 (11.06,12.37) |
| 21 | 13.80 (13.11,14.52) | 14.51 (13.82,15.23) | 14.76 (14.04,15.52) |
| 22 | 17.15 (16.30,18.05) | 17.70 (16.83,18.61) | 18.31 (17.43,19.24) |
| 23 | 21.01 (19.96,22.12) | 21.37 (20.29,22.50) | 22.36 (21.29,23.49) |
| 24 | 25.38 (24.16,26.65) | 25.57 (24.33,26.87) | 26.90 (25.68,28.17) |
| 25 | 30.22 (28.88,31.63) | 30.33 (28.97,31.76) | 31.89 (30.55,33.28) |
| 26 | 35.52 (33.98,37.13) | 35.67 (34.12,37.29) | 37.29 (35.77,38.87) |
| 27 | 41.21 (39.37,43.14) | 41.59 (39.74,43.52) | 43.03 (41.23,44.91) |
| 28 | 47.23 (45.06,49.49) | 48.07 (45.87,50.38) | 49.03 (46.91,51.24) |
| 29 | 53.48 (51.15,55.92) | 55.10 (52.67,57.64) | 55.20 (52.89,57.60) |
| 30 | 59.90 (57.52,62.37) | 62.58 (60.04,65.23) | 61.48 (59.12,63.94) |
| 31 | 66.42 (63.80,69.14) | 70.33 (67.48,73.30) | 67.93 (65.36,70.62) |
| 32 | 73.00 (69.87,76.27) | 78.08 (74.65,81.66) | 74.65 (71.62,77.80) |
| 33 | 79.63 (76.07,83.35) | 85.49 (81.60,89.57) | 81.77 (78.34,85.36) |
| 34 | 86.31 (82.62,90.16) | 92.28 (88.31,96.43) | 89.49 (85.88,93.26) |
| 35 | 93.13 (89.02,97.43) | 98.66 (94.42,103.08) | 97.81 (93.82,101.96) |
| 36 | 100.25 (95.25,105.51) | 105.16 (100.14,110.43) | 106.61 (101.81,111.64) |
| 37 | 107.87 (102.06,114.00) | 112.49 (106.70,118.60) | 115.73 (110.15,121.59) |
| 38 | 116.26 (109.35,123.61) | 121.57 (115.15,128.35) | 124.97 (118.82,131.43) |
| 39 | 125.77 (113.99,138.76) | 133.61 (123.98,143.98) | 134.04 (125.26,143.44) |
| 40 | 136.85 (113.25,165.36) | 150.30 (130.24,173.46) | 142.64 (125.58,162.02) |

© 2025 Clayton PK et al. *JAMA Network Open*.

**eTable 21.** Means of Estimated Fetal Weight by Gestational Week in Fetuses of Women with PHDI (T1), PHDI (T2), and PHDI (T3) among the Standard Population in the NICHD Fetal Growth Studies, 2009-2013.

| | Estimated Fetal Weight, g (95% CI) | | |
|---|---|---|---|
| GA | T1 | T2 | T3 |
| 15 | 108 (105,112) | 110 (106,114) | 111 (108,114) |
| 16 | 139 (137,142) | 141 (138,144) | 141 (138,143) |
| 17 | 177 (174,179) | 177 (175,180) | 176 (174,178) |
| 18 | 220 (217,223) | 221 (218,224) | 218 (215,220) |
| 19 | 271 (267,274) | 271 (268,275) | 267 (264,270) |
| 20 | 329 (324,333) | 330 (326,334) | 324 (320,328) |
| 21 | 394 (390,400) | 397 (392,402) | 391 (386,395) |
| 22 | 469 (463,475) | 473 (467,478) | 467 (461,472) |
| 23 | 553 (546,560) | 559 (552,565) | 553 (547,559) |
| 24 | 648 (640,656) | 655 (648,663) | 650 (643,658) |
| 25 | 754 (745,764) | 764 (755,773) | 759 (751,768) |
| 26 | 874 (863,884) | 885 (875,895) | 881 (871,890) |
| 27 | 1006 (994,1017) | 1019 (1008,1030) | 1015 (1004,1026) |
| 28 | 1151 (1138,1164) | 1167 (1154,1179) | 1163 (1150,1175) |
| 29 | 1309 (1294,1325) | 1328 (1314,1343) | 1324 (1310,1338) |
| 30 | 1480 (1463,1498) | 1504 (1487,1520) | 1498 (1482,1514) |
| 31 | 1662 (1643,1682) | 1693 (1674,1712) | 1686 (1668,1705) |
| 32 | 1855 (1833,1876) | 1895 (1875,1916) | 1887 (1867,1907) |
| 33 | 2056 (2031,2080) | 2108 (2085,2132) | 2098 (2075,2121) |
| 34 | 2263 (2235,2291) | 2329 (2302,2356) | 2315 (2289,2342) |
| 35 | 2475 (2444,2506) | 2553 (2523,2583) | 2535 (2506,2565) |
| 36 | 2688 (2654,2722) | 2775 (2742,2808) | 2752 (2721,2784) |
| 37 | 2900 (2862,2939) | 2992 (2955,3030) | 2966 (2930,3001) |
| 38 | 3109 (3067,3152) | 3200 (3158,3242) | 3178 (3139,3218) |
| 39 | 3313 (3262,3365) | 3396 (3349,3444) | 3396 (3350,3441) |
| 40 | 3510 (3412,3611) | 3578 (3497,3661) | 3623 (3550,3698) |

© 2025 Clayton PK et al. *JAMA Network Open*.

**eTable 22.** Means of Head Circumference by Gestational Week in Fetuses of Women with PHDI (T1), PHDI (T2), and PHDI (T3) among the Standard Population in the NICHD Fetal Growth Studies, 2009-2013.

| | Mean Head Circumference, mm (95% CI) | | |
|---|---|---|---|
| GA | T1 | T2 | T3 |
| 15 | 110.3 (109.0,111.7) | 110.5 (109.1,112.0) | 110.5 (109.4,111.6) |
| 16 | 122.9 (121.9,123.8) | 123.2 (122.3,124.2) | 122.8 (122.0,123.6) |
| 17 | 135.5 (134.7,136.2) | 136.0 (135.2,136.7) | 135.2 (134.6,135.8) |
| 18 | 148.0 (147.3,148.7) | 148.5 (147.8,149.2) | 147.5 (146.8,148.2) |
| 19 | 160.3 (159.5,161.1) | 160.8 (160.1,161.6) | 159.6 (158.9,160.3) |
| 20 | 172.4 (171.5,173.2) | 172.8 (172.1,173.6) | 171.5 (170.7,172.2) |
| 21 | 184.1 (183.3,185.0) | 184.5 (183.7,185.3) | 183.1 (182.3,183.8) |
| 22 | 195.6 (194.8,196.4) | 195.8 (195.0,196.6) | 194.4 (193.7,195.2) |
| 23 | 206.9 (206.0,207.7) | 206.9 (206.1,207.7) | 205.6 (204.8,206.4) |
| 24 | 217.9 (217.0,218.9) | 217.9 (217.0,218.7) | 216.7 (215.9,217.5) |
| 25 | 228.9 (228.0,229.9) | 228.8 (227.9,229.7) | 227.8 (226.9,228.6) |
| 26 | 239.8 (238.9,240.7) | 239.7 (238.8,240.5) | 238.8 (237.9,239.6) |
| 27 | 250.4 (249.5,251.3) | 250.3 (249.4,251.2) | 249.6 (248.7,250.4) |
| 28 | 260.6 (259.7,261.6) | 260.6 (259.7,261.5) | 260.0 (259.2,260.9) |
| 29 | 270.4 (269.4,271.4) | 270.4 (269.5,271.4) | 270.0 (269.1,270.9) |
| 30 | 279.6 (278.6,280.6) | 279.6 (278.7,280.6) | 279.3 (278.4,280.2) |
| 31 | 288.1 (287.0,289.1) | 288.1 (287.1,289.0) | 287.8 (286.9,288.7) |
| 32 | 295.8 (294.7,296.8) | 295.7 (294.7,296.6) | 295.5 (294.6,296.5) |
| 33 | 302.7 (301.6,303.7) | 302.6 (301.6,303.6) | 302.5 (301.5,303.4) |
| 34 | 308.8 (307.7,310.0) | 308.8 (307.8,309.9) | 308.7 (307.7,309.8) |
| 35 | 314.2 (313.1,315.4) | 314.6 (313.5,315.7) | 314.4 (313.4,315.5) |
| 36 | 318.9 (317.7,320.0) | 320.0 (319.0,321.1) | 319.7 (318.6,320.7) |
| 37 | 323.0 (321.7,324.2) | 324.9 (323.7,326.1) | 324.4 (323.3,325.6) |
| 38 | 326.7 (325.4,328.0) | 328.9 (327.6,330.2) | 328.7 (327.5,329.9) |
| 39 | 330.3 (328.8,331.9) | 331.6 (330.2,333.0) | 332.4 (331.1,333.7) |
| 40 | 334.1 (330.9,337.3) | 332.7 (330.2,335.2) | 335.5 (333.2,337.7) |

© 2025 Clayton PK et al. *JAMA Network Open*.

**eTable 23**. Means of Abdominal Circumference by Gestational Week in Fetuses of Women with PHDI (T1), PHDI (T2), and PHDI (T3) among the Standard Population in the NICHD Fetal Growth Studies, 2009-2013.

| | Mean Abdominal Circumference, mm (95% CI) | | |
|---|---|---|---|
| GA | T1 | T2 | T3 |
| 15 | 110.3 (109.0,111.7) | 110.5 (109.1,112.0) | 110.5 (109.4,111.6) |
| 16 | 122.9 (121.9,123.8) | 123.2 (122.3,124.2) | 122.8 (122.0,123.6) |
| 17 | 135.5 (134.7,136.2) | 136.0 (135.2,136.7) | 135.2 (134.6,135.8) |
| 18 | 148.0 (147.3,148.7) | 148.5 (147.8,149.2) | 147.5 (146.8,148.2) |
| 19 | 160.3 (159.5,161.1) | 160.8 (160.1,161.6) | 159.6 (158.9,160.3) |
| 20 | 172.4 (171.5,173.2) | 172.8 (172.1,173.6) | 171.5 (170.7,172.2) |
| 21 | 184.1 (183.3,185.0) | 184.5 (183.7,185.3) | 183.1 (182.3,183.8) |
| 22 | 195.6 (194.8,196.4) | 195.8 (195.0,196.6) | 194.4 (193.7,195.2) |
| 23 | 206.9 (206.0,207.7) | 206.9 (206.1,207.7) | 205.6 (204.8,206.4) |
| 24 | 217.9 (217.0,218.9) | 217.9 (217.0,218.7) | 216.7 (215.9,217.5) |
| 25 | 228.9 (228.0,229.9) | 228.8 (227.9,229.7) | 227.8 (226.9,228.6) |
| 26 | 239.8 (238.9,240.7) | 239.7 (238.8,240.5) | 238.8 (237.9,239.6) |
| 27 | 250.4 (249.5,251.3) | 250.3 (249.4,251.2) | 249.6 (248.7,250.4) |
| 28 | 260.6 (259.7,261.6) | 260.6 (259.7,261.5) | 260.0 (259.2,260.9) |
| 29 | 270.4 (269.4,271.4) | 270.4 (269.5,271.4) | 270.0 (269.1,270.9) |
| 30 | 279.6 (278.6,280.6) | 279.6 (278.7,280.6) | 279.3 (278.4,280.2) |
| 31 | 288.1 (287.0,289.1) | 288.1 (287.1,289.0) | 287.8 (286.9,288.7) |
| 32 | 295.8 (294.7,296.8) | 295.7 (294.7,296.6) | 295.5 (294.6,296.5) |
| 33 | 302.7 (301.6,303.7) | 302.6 (301.6,303.6) | 302.5 (301.5,303.4) |
| 34 | 308.8 (307.7,310.0) | 308.8 (307.8,309.9) | 308.7 (307.7,309.8) |
| 35 | 314.2 (313.1,315.4) | 314.6 (313.5,315.7) | 314.4 (313.4,315.5) |
| 36 | 318.9 (317.7,320.0) | 320.0 (319.0,321.1) | 319.7 (318.6,320.7) |
| 37 | 323.0 (321.7,324.2) | 324.9 (323.7,326.1) | 324.4 (323.3,325.6) |
| 38 | 326.7 (325.4,328.0) | 328.9 (327.6,330.2) | 328.7 (327.5,329.9) |
| 39 | 330.3 (328.8,331.9) | 331.6 (330.2,333.0) | 332.4 (331.1,333.7) |
| 40 | 334.1 (330.9,337.3) | 332.7 (330.2,335.2) | 335.5 (333.2,337.7) |

© 2025 Clayton PK et al. *JAMA Network Open*.

**eTable 24.** Means of Average Humerus Length by Gestational Week in Fetuses of Women with PHDI (T1), PHDI (T2), and PHDI (T3) among the Standard Population in the NICHD Fetal Growth Studies, 2009-2013.

| | Mean Humerus Length, mm (95% CI) | | |
|---|---|---|---|
| GA | T1 | T2 | T3 |
| 15 | 16.7 (16.4,16.9) | 16.7 (16.4,16.9) | 16.5 (16.3,16.7) |
| 16 | 19.7 (19.5,19.9) | 19.7 (19.5,19.9) | 19.5 (19.3,19.6) |
| 17 | 22.7 (22.5,22.8) | 22.7 (22.5,22.8) | 22.4 (22.3,22.6) |
| 18 | 25.6 (25.5,25.8) | 25.6 (25.4,25.8) | 25.3 (25.2,25.5) |
| 19 | 28.5 (28.3,28.6) | 28.4 (28.2,28.6) | 28.1 (28.0,28.3) |
| 20 | 31.1 (30.9,31.3) | 31.1 (30.9,31.3) | 30.8 (30.6,30.9) |
| 21 | 33.6 (33.4,33.8) | 33.5 (33.3,33.7) | 33.2 (33.0,33.4) |
| 22 | 35.9 (35.7,36.1) | 35.8 (35.6,36.0) | 35.5 (35.3,35.7) |
| 23 | 38.0 (37.8,38.2) | 37.9 (37.7,38.1) | 37.6 (37.4,37.8) |
| 24 | 40.0 (39.8,40.2) | 40.0 (39.7,40.2) | 39.7 (39.5,39.9) |
| 25 | 42.0 (41.7,42.2) | 41.9 (41.7,42.2) | 41.7 (41.5,41.9) |
| 26 | 43.9 (43.6,44.1) | 43.9 (43.6,44.1) | 43.6 (43.4,43.8) |
| 27 | 45.7 (45.5,46.0) | 45.7 (45.5,46.0) | 45.5 (45.3,45.7) |
| 28 | 47.5 (47.3,47.8) | 47.6 (47.3,47.8) | 47.4 (47.2,47.6) |
| 29 | 49.3 (49.0,49.5) | 49.3 (49.1,49.5) | 49.1 (48.9,49.4) |
| 30 | 50.9 (50.7,51.2) | 51.0 (50.8,51.2) | 50.8 (50.6,51.0) |
| 31 | 52.5 (52.3,52.8) | 52.6 (52.4,52.8) | 52.4 (52.2,52.6) |
| 32 | 54.0 (53.8,54.3) | 54.2 (53.9,54.4) | 53.9 (53.7,54.1) |
| 33 | 55.5 (55.2,55.7) | 55.6 (55.4,55.9) | 55.3 (55.1,55.6) |
| 34 | 56.8 (56.6,57.1) | 57.1 (56.8,57.3) | 56.7 (56.5,57.0) |
| 35 | 58.2 (57.9,58.4) | 58.4 (58.2,58.7) | 58.0 (57.8,58.3) |
| 36 | 59.4 (59.2,59.7) | 59.7 (59.5,60.0) | 59.3 (59.1,59.6) |
| 37 | 60.7 (60.4,61.0) | 60.9 (60.7,61.2) | 60.6 (60.3,60.8) |
| 38 | 61.9 (61.6,62.2) | 62.0 (61.7,62.3) | 61.7 (61.5,62.0) |
| 39 | 63.0 (62.7,63.4) | 62.8 (62.5,63.1) | 62.8 (62.5,63.1) |
| 40 | 64.2 (63.5,64.8) | 63.4 (62.8,63.9) | 63.6 (63.1,64.1) |

© 2025 Clayton PK et al. *JAMA Network Open*.

**eTable 25.** Means of Average Femur Length by Gestational Week in Fetuses of Women with PHDI (T1), PHDI (T2), and PHDI (T3) among the Standard Population in the NICHD Fetal Growth Studies, 2009-2013.

| | Mean Femur Length, mm (95% CI) | | |
|---|---|---|---|
| GA | T1 | T2 | T3 |
| 15 | 16.1 (15.8,16.4) | 16.3 (16.0,16.6) | 16.3 (16.1,16.5) |
| 16 | 19.3 (19.1,19.5) | 19.4 (19.2,19.6) | 19.3 (19.2,19.5) |
| 17 | 22.5 (22.4,22.7) | 22.6 (22.4,22.8) | 22.5 (22.3,22.6) |
| 18 | 25.8 (25.6,25.9) | 25.8 (25.6,25.9) | 25.6 (25.4,25.8) |
| 19 | 28.9 (28.7,29.1) | 28.9 (28.7,29.1) | 28.7 (28.5,28.9) |
| 20 | 31.9 (31.7,32.1) | 31.9 (31.7,32.1) | 31.7 (31.5,31.9) |
| 21 | 34.8 (34.5,35.0) | 34.8 (34.6,35.0) | 34.5 (34.3,34.7) |
| 22 | 37.4 (37.2,37.7) | 37.5 (37.3,37.8) | 37.2 (37.0,37.4) |
| 23 | 40.0 (39.7,40.2) | 40.1 (39.9,40.4) | 39.8 (39.6,40.0) |
| 24 | 42.4 (42.2,42.7) | 42.6 (42.4,42.9) | 42.3 (42.1,42.6) |
| 25 | 44.9 (44.6,45.1) | 45.1 (44.8,45.3) | 44.8 (44.6,45.0) |
| 26 | 47.3 (47.0,47.5) | 47.4 (47.2,47.7) | 47.2 (47.0,47.5) |
| 27 | 49.7 (49.4,49.9) | 49.8 (49.5,50.0) | 49.6 (49.4,49.8) |
| 28 | 52.0 (51.7,52.2) | 52.0 (51.8,52.3) | 51.9 (51.7,52.1) |
| 29 | 54.2 (54.0,54.5) | 54.3 (54.0,54.5) | 54.1 (53.9,54.4) |
| 30 | 56.4 (56.1,56.6) | 56.4 (56.2,56.7) | 56.3 (56.1,56.5) |
| 31 | 58.4 (58.1,58.7) | 58.5 (58.3,58.8) | 58.4 (58.1,58.6) |
| 32 | 60.3 (60.0,60.6) | 60.5 (60.3,60.8) | 60.3 (60.1,60.6) |
| 33 | 62.1 (61.8,62.4) | 62.5 (62.3,62.8) | 62.2 (62.0,62.5) |
| 34 | 63.9 (63.6,64.2) | 64.4 (64.1,64.7) | 64.0 (63.7,64.3) |
| 35 | 65.6 (65.3,65.9) | 66.2 (65.9,66.5) | 65.7 (65.5,66.0) |
| 36 | 67.4 (67.1,67.7) | 67.9 (67.6,68.1) | 67.4 (67.1,67.7) |
| 37 | 69.1 (68.8,69.4) | 69.4 (69.1,69.7) | 69.0 (68.7,69.2) |
| 38 | 70.5 (70.2,70.9) | 70.8 (70.5,71.2) | 70.5 (70.1,70.8) |
| 39 | 71.6 (71.1,72.0) | 72.1 (71.7,72.5) | 71.9 (71.5,72.2) |
| 40 | 71.9 (71.1,72.7) | 73.2 (72.6,73.9) | 73.2 (72.6,73.8) |

© 2025 Clayton PK et al. *JAMA Network Open*.

**eTable 26**. Means of Fractional Arm Volume by Gestational Week in Fetuses of Women with PHDI (T1), PHDI (T2), and PHDI (T3) among the Standard Population in the NICHD Fetal Growth Studies, 2009-2013.

| | Mean Fractional Arm Volume, cm3 (95% CI) | | |
|---|---|---|---|
| GA | T1 | T2 | T3 |
| 15 | 0.47 (0.42,0.53) | 0.50 (0.45,0.56) | 0.50 (0.46,0.54) |
| 16 | 0.71 (0.67,0.75) | 0.74 (0.69,0.78) | 0.74 (0.71,0.77) |
| 17 | 1.02 (0.99,1.06) | 1.05 (1.01,1.08) | 1.05 (1.01,1.08) |
| 18 | 1.42 (1.37,1.46) | 1.43 (1.39,1.48) | 1.42 (1.38,1.47) |
| 19 | 1.90 (1.84,1.96) | 1.90 (1.85,1.96) | 1.88 (1.82,1.93) |
| 20 | 2.46 (2.39,2.53) | 2.45 (2.39,2.52) | 2.41 (2.35,2.47) |
| 21 | 3.10 (3.02,3.18) | 3.09 (3.01,3.16) | 3.01 (2.94,3.09) |
| 22 | 3.81 (3.71,3.91) | 3.79 (3.70,3.89) | 3.70 (3.62,3.79) |
| 23 | 4.58 (4.46,4.70) | 4.57 (4.46,4.69) | 4.48 (4.38,4.59) |
| 24 | 5.40 (5.27,5.54) | 5.42 (5.29,5.55) | 5.36 (5.24,5.48) |
| 25 | 6.29 (6.15,6.44) | 6.35 (6.20,6.49) | 6.33 (6.20,6.46) |
| 26 | 7.25 (7.09,7.42) | 7.35 (7.19,7.51) | 7.40 (7.25,7.54) |
| 27 | 8.32 (8.12,8.52) | 8.45 (8.26,8.64) | 8.56 (8.39,8.74) |
| 28 | 9.52 (9.29,9.75) | 9.67 (9.45,9.90) | 9.84 (9.63,10.04) |
| 29 | 10.91 (10.66,11.16) | 11.06 (10.82,11.31) | 11.22 (11.00,11.45) |
| 30 | 12.53 (12.26,12.80) | 12.63 (12.37,12.90) | 12.73 (12.50,12.97) |
| 31 | 14.36 (14.04,14.68) | 14.40 (14.10,14.72) | 14.38 (14.11,14.67) |
| 32 | 16.37 (15.97,16.77) | 16.38 (16.00,16.78) | 16.20 (15.85,16.55) |
| 33 | 18.51 (18.05,18.99) | 18.57 (18.12,19.03) | 18.21 (17.80,18.63) |
| 34 | 20.71 (20.20,21.24) | 20.95 (20.45,21.45) | 20.45 (20.00,20.91) |
| 35 | 22.95 (22.32,23.60) | 23.46 (22.87,24.08) | 22.9 (22.39,23.43) |
| 36 | 25.22 (24.42,26.05) | 26.03 (25.26,26.82) | 25.54 (24.90,26.19) |
| 37 | 27.53 (26.60,28.49) | 28.52 (27.61,29.47) | 28.29 (27.53,29.07) |
| 38 | 29.89 (28.80,31.01) | 30.79 (29.76,31.85) | 31.07 (30.18,32.00) |
| 39 | 32.33 (30.40,34.37) | 32.64 (31.13,34.22) | 33.78 (32.50,35.10) |
| 40 | 34.89 (30.93,39.36) | 33.91 (31.03,37.04) | 36.26 (33.93,38.74) |

© 2025 Clayton PK et al. *JAMA Network Open*.

**eTable 27**. Means of Fractional Lean Arm Volume by Gestational Week in Fetuses of Women with PHDI (T1), PHDI (T2), and PHDI (T3) among the Standard Population in the NICHD Fetal Growth Studies, 2009-2013.

| | Mean Fractional Lean Arm Volume, $cm^3$ (95% CI) | | |
|---|---|---|---|
| GA | T1 | T2 | T3 |
| 15 | 0.21 (0.19,0.24) | 0.22 (0.20,0.25) | 0.22 (0.20,0.24) |
| 16 | 0.34 (0.32,0.36) | 0.35 (0.33,0.37) | 0.35 (0.33,0.37) |
| 17 | 0.51 (0.49,0.53) | 0.52 (0.50,0.54) | 0.52 (0.50,0.53) |
| 18 | 0.73 (0.70,0.75) | 0.73 (0.70,0.75) | 0.72 (0.70,0.75) |
| 19 | 0.98 (0.95,1.01) | 0.98 (0.95,1.01) | 0.97 (0.94,1.00) |
| 20 | 1.26 (1.22,1.30) | 1.26 (1.23,1.30) | 1.25 (1.21,1.28) |
| 21 | 1.57 (1.53,1.61) | 1.58 (1.54,1.61) | 1.55 (1.52,1.59) |
| 22 | 1.90 (1.86,1.95) | 1.91 (1.87,1.96) | 1.88 (1.84,1.93) |
| 23 | 2.25 (2.20,2.31) | 2.27 (2.21,2.32) | 2.24 (2.19,2.29) |
| 24 | 2.63 (2.57,2.70) | 2.65 (2.58,2.71) | 2.63 (2.57,2.69) |
| 25 | 3.04 (2.98,3.11) | 3.05 (2.98,3.12) | 3.05 (2.99,3.12) |
| 26 | 3.49 (3.41,3.56) | 3.48 (3.40,3.55) | 3.51 (3.44,3.58) |
| 27 | 3.97 (3.88,4.05) | 3.94 (3.85,4.02) | 4.00 (3.92,4.08) |
| 28 | 4.48 (4.38,4.59) | 4.43 (4.34,4.53) | 4.53 (4.43,4.62) |
| 29 | 5.05 (4.94,5.17) | 4.99 (4.88,5.09) | 5.09 (4.99,5.19) |
| 30 | 5.68 (5.57,5.80) | 5.60 (5.50,5.71) | 5.69 (5.58,5.80) |
| 31 | 6.38 (6.25,6.51) | 6.28 (6.16,6.41) | 6.33 (6.21,6.45) |
| 32 | 7.15 (6.99,7.32) | 7.01 (6.85,7.17) | 7.02 (6.87,7.17) |
| 33 | 8.01 (7.82,8.21) | 7.77 (7.58,7.95) | 7.76 (7.59,7.93) |
| 34 | 8.96 (8.75,9.18) | 8.54 (8.35,8.74) | 8.55 (8.37,8.74) |
| 35 | 9.97 (9.71,10.23) | 9.32 (9.09,9.54) | 9.40 (9.20,9.61) |
| 36 | 10.97 (10.64,11.31) | 10.10 (9.81,10.38) | 10.30 (10.05,10.55) |
| 37 | 11.87 (11.49,12.27) | 10.88 (10.54,11.22) | 11.24 (10.94,11.55) |
| 38 | 12.59 (12.14,13.05) | 11.66 (11.29,12.04) | 12.21 (11.87,12.57) |
| 39 | 12.99 (12.24,13.79) | 12.45 (11.91,13.01) | 13.20 (12.72,13.71) |
| 40 | 13.00 (11.57,14.60) | 13.25 (12.21,14.37) | 14.20 (13.30,15.15) |

© 2025 Clayton PK et al. *JAMA Network Open*.

**eTable 28**. Means of Fractional Fat Arm Volume by Gestational Week in Fetuses of Women with PHDI (T1), PHDI (T2), and PHDI (T3) among the Standard Population in the NICHD Fetal Growth Studies, 2009-2013.

| | Mean Fractional Fat Arm Volume, $cm^3$ (95% CI) | | |
|---|---|---|---|
| GA | T1 | T2 | T3 |
| 15 | 0.25 (0.22,0.29) | 0.27 (0.24,0.31) | 0.25 (0.23,0.28) |
| 16 | 0.37 (0.34,0.40) | 0.39 (0.36,0.42) | 0.37 (0.35,0.39) |
| 17 | 0.53 (0.50,0.55) | 0.53 (0.51,0.56) | 0.52 (0.50,0.54) |
| 18 | 0.72 (0.69,0.75) | 0.72 (0.69,0.74) | 0.70 (0.68,0.73) |
| 19 | 0.95 (0.91,0.99) | 0.94 (0.90,0.97) | 0.92 (0.89,0.95) |
| 20 | 1.22 (1.17,1.26) | 1.20 (1.16,1.24) | 1.18 (1.14,1.22) |
| 21 | 1.52 (1.47,1.57) | 1.50 (1.46,1.55) | 1.48 (1.44,1.52) |
| 22 | 1.87 (1.81,1.92) | 1.85 (1.80,1.91) | 1.82 (1.77,1.88) |
| 23 | 2.25 (2.19,2.32) | 2.26 (2.19,2.32) | 2.22 (2.16,2.29) |
| 24 | 2.69 (2.61,2.77) | 2.71 (2.64,2.80) | 2.68 (2.61,2.76) |
| 25 | 3.18 (3.10,3.27) | 3.24 (3.15,3.33) | 3.22 (3.14,3.30) |
| 26 | 3.74 (3.64,3.84) | 3.82 (3.73,3.92) | 3.83 (3.74,3.92) |
| 27 | 4.37 (4.26,4.49) | 4.48 (4.37,4.60) | 4.52 (4.41,4.63) |
| 28 | 5.10 (4.96,5.24) | 5.23 (5.09,5.37) | 5.30 (5.16,5.43) |
| 29 | 5.95 (5.79,6.11) | 6.06 (5.91,6.22) | 6.15 (6.00,6.30) |
| 30 | 6.93 (6.77,7.10) | 7.00 (6.84,7.17) | 7.08 (6.92,7.25) |
| 31 | 8.05 (7.85,8.25) | 8.07 (7.87,8.27) | 8.12 (7.93,8.31) |
| 32 | 9.27 (9.01,9.53) | 9.28 (9.03,9.53) | 9.26 (9.02,9.50) |
| 33 | 10.54 (10.24,10.85) | 10.65 (10.35,10.96) | 10.54 (10.26,10.83) |
| 34 | 11.81 (11.48,12.15) | 12.23 (11.90,12.57) | 11.99 (11.68,12.31) |
| 35 | 13.09 (12.69,13.50) | 13.96 (13.56,14.36) | 13.60 (13.24,13.97) |
| 36 | 14.43 (13.92,14.95) | 15.76 (15.25,16.30) | 15.33 (14.88,15.79) |
| 37 | 15.90 (15.30,16.53) | 17.52 (16.89,18.17) | 17.12 (16.58,17.68) |
| 38 | 17.63 (16.90,18.39) | 19.06 (18.34,19.81) | 18.89 (18.26,19.55) |
| 39 | 19.76 (18.43,21.18) | 20.19 (19.14,21.30) | 20.53 (19.63,21.48) |
| 40 | 22.51 (19.64,25.80) | 20.71 (18.73,22.89) | 21.91 (20.23,23.73) |

© 2025 Clayton PK et al. *JAMA Network Open*.

**eTable 29.** Means of Maximum Arm Subcutaneous Tissue Thickness (SCTT) by Gestational Week in Fetuses of Women with PHDI (T1), PHDI (T2), and PHDI (T3) among the Standard Population in the NICHD Fetal Growth Studies, 2009-2013.

| | Mean Maximum Arm SCTT, cm (95% CI) | | |
|---|---|---|---|
| GA | T1 | T2 | T3 |
| 15 | 0.15 (0.13,0.17) | 0.17 (0.15,0.20) | 0.16 (0.15,0.18) |
| 16 | 0.18 (0.17,0.20) | 0.19 (0.17,0.20) | 0.18 (0.17,0.20) |
| 17 | 0.21 (0.20,0.22) | 0.21 (0.20,0.22) | 0.21 (0.20,0.22) |
| 18 | 0.24 (0.23,0.25) | 0.23 (0.22,0.24) | 0.23 (0.22,0.24) |
| 19 | 0.26 (0.25,0.28) | 0.25 (0.24,0.26) | 0.25 (0.24,0.26) |
| 20 | 0.28 (0.27,0.30) | 0.27 (0.26,0.28) | 0.28 (0.27,0.29) |
| 21 | 0.30 (0.29,0.32) | 0.30 (0.29,0.31) | 0.30 (0.29,0.31) |
| 22 | 0.32 (0.31,0.34) | 0.33 (0.31,0.34) | 0.32 (0.31,0.34) |
| 23 | 0.34 (0.33,0.36) | 0.35 (0.34,0.37) | 0.35 (0.33,0.36) |
| 24 | 0.36 (0.35,0.38) | 0.38 (0.37,0.39) | 0.37 (0.36,0.38) |
| 25 | 0.39 (0.37,0.40) | 0.41 (0.39,0.42) | 0.39 (0.38,0.41) |
| 26 | 0.41 (0.40,0.43) | 0.43 (0.42,0.45) | 0.42 (0.40,0.43) |
| 27 | 0.44 (0.43,0.46) | 0.46 (0.44,0.48) | 0.44 (0.43,0.45) |
| 28 | 0.48 (0.46,0.50) | 0.49 (0.47,0.51) | 0.47 (0.45,0.48) |
| 29 | 0.51 (0.49,0.53) | 0.52 (0.50,0.54) | 0.50 (0.48,0.51) |
| 30 | 0.55 (0.53,0.57) | 0.56 (0.54,0.58) | 0.53 (0.52,0.55) |
| 31 | 0.59 (0.57,0.61) | 0.59 (0.57,0.62) | 0.57 (0.56,0.59) |
| 32 | 0.62 (0.60,0.65) | 0.63 (0.61,0.66) | 0.62 (0.60,0.64) |
| 33 | 0.66 (0.63,0.69) | 0.68 (0.65,0.70) | 0.66 (0.64,0.69) |
| 34 | 0.70 (0.67,0.72) | 0.72 (0.69,0.75) | 0.71 (0.69,0.74) |
| 35 | 0.73 (0.70,0.76) | 0.76 (0.73,0.79) | 0.76 (0.73,0.79) |
| 36 | 0.77 (0.73,0.81) | 0.80 (0.76,0.84) | 0.81 (0.78,0.84) |
| 37 | 0.81 (0.77,0.86) | 0.83 (0.79,0.88) | 0.85 (0.81,0.89) |
| 38 | 0.87 (0.82,0.92) | 0.86 (0.82,0.91) | 0.89 (0.84,0.93) |
| 39 | 0.93 (0.85,1.02) | 0.88 (0.82,0.94) | 0.91 (0.85,0.97) |
| 40 | 1.02 (0.86,1.22) | 0.89 (0.79,1.01) | 0.91 (0.82,1.02) |

© 2025 Clayton PK et al. *JAMA Network Open*.

**eTable 30.** Means of Abdominal Area by Gestational Week in Fetuses of Women with PHDI (T1), PHDI (T2), and PHDI (T3) among the Standard Population in the NICHD Fetal Growth Studies, 2009-2013.

| | Mean Abdominal Area, $mm^2$ (95% CI) | | |
|---|---|---|---|
| GA | T1 | T2 | T3 |
| 15 | 658.52 (631.74,686.43) | 661.81 (632.05,692.98) | 699.31 (676.26,723.14) |
| 16 | 853.99 (833.40,875.08) | 851.25 (828.30,874.82) | 878.33 (860.78,896.24) |
| 17 | 1074.35 (1057.05,1091.93) | 1066.96 (1048.70,1085.55) | 1082.16 (1066.62,1097.92) |
| 18 | 1315.46 (1296.07,1335.14) | 1306.35 (1287.57,1325.40) | 1309.74 (1291.81,1327.91) |
| 19 | 1572.81 (1549.19,1596.79) | 1566.11 (1543.74,1588.82) | 1559.4 (1537.75,1581.36) |
| 20 | 1842.33 (1815.49,1869.57) | 1842.83 (1817.27,1868.74) | 1829.08 (1804.60,1853.89) |
| 21 | 2121.19 (2092.48,2150.30) | 2133.44 (2105.93,2161.32) | 2116.53 (2090.21,2143.18) |
| 22 | 2408.46 (2377.56,2439.76) | 2435.88 (2406.17,2465.95) | 2419.64 (2391.11,2448.52) |
| 23 | 2705.66 (2670.55,2741.23) | 2749.46 (2715.64,2783.70) | 2736.74 (2704.31,2769.55) |
| 24 | 3017.24 (2976.69,3058.34) | 3075.37 (3036.23,3115.01) | 3066.82 (3029.56,3104.54) |
| 25 | 3349.17 (3304.60,3394.35) | 3416.08 (3373.13,3459.59) | 3409.63 (3368.84,3450.91) |
| 26 | 3702.51 (3655.75,3749.86) | 3772.2 (3727.38,3817.56) | 3765.07 (3722.36,3808.26) |
| 27 | 4076.50 (4027.76,4125.82) | 4144.13 (4097.64,4191.14) | 4133.70 (4089.17,4178.73) |
| 28 | 4470.05 (4417.54,4523.19) | 4532.93 (4482.83,4583.59) | 4517.13 (4469.07,4565.70) |
| 29 | 4881.74 (4823.32,4940.86) | 4940.48 (4884.51,4997.09) | 4918.09 (4864.61,4972.16) |
| 30 | 5309.75 (5245.38,5374.91) | 5369.58 (5307.67,5432.22) | 5340.69 (5281.70,5400.33) |
| 31 | 5751.93 (5683.85,5820.83) | 5823.69 (5758.19,5889.94) | 5789.98 (5727.38,5853.26) |
| 32 | 6205.78 (6133.75,6278.66) | 6300.69 (6231.52,6370.62) | 6263.52 (6197.12,6330.63) |
| 33 | 6668.49 (6588.28,6749.68) | 6792.04 (6714.97,6869.99) | 6750.26 (6676.34,6825.00) |
| 34 | 7136.93 (7046.22,7228.82) | 7286.46 (7199.01,7374.97) | 7235.49 (7151.80,7320.17) |
| 35 | 7607.70 (7510.19,7706.48) | 7769.97 (7675.84,7865.25) | 7701.00 (7610.72,7792.34) |
| 36 | 8077.08 (7975.76,8179.68) | 8227.83 (8130.38,8326.46) | 8127.64 (8034.83,8221.52) |
| 37 | 8540.01 (8424.47,8657.13) | 8672.12 (8560.68,8785.01) | 8529.96 (8427.45,8633.73) |
| 38 | 8990.15 (8860.77,9121.43) | 9142.21 (9016.34,9269.84) | 8956.18 (8841.05,9072.81) |
| 39 | 9420.64 (9261.77,9582.24) | 9687.33 (9543.63,9833.19) | 9465.75 (9332.07,9601.34) |
| 40 | 9824.24 (9485.75,10174.81) | 10368.72 (10075.54,10670.44) | 10132.27 (9881.56,10389.35) |

© 2025 Clayton PK et al. *JAMA Network Open*.

**eTable 31.** Means of Maximum Abdominal Subcutaneous Tissue Thickness (SCTT) by Gestational Week in Fetuses of Women with PHDI (T1), PHDI (T2), and PHDI (T3) among the Standard Population in the NICHD Fetal Growth Studies, 2009-2013.

| | Mean Maximum Abdominal SCTT, cm (95% CI) | | |
|---|---|---|---|
| GA | T1 | T2 | T3 |
| 15 | 1.31 (1.16,1.47) | 1.38 (1.21,1.57) | 1.43 (1.30,1.57) |
| 16 | 1.48 (1.38,1.58) | 1.55 (1.43,1.67) | 1.56 (1.47,1.64) |
| 17 | 1.63 (1.56,1.71) | 1.70 (1.63,1.79) | 1.67 (1.61,1.74) |
| 18 | 1.76 (1.70,1.84) | 1.85 (1.78,1.92) | 1.78 (1.72,1.85) |
| 19 | 1.88 (1.80,1.95) | 1.97 (1.90,2.05) | 1.89 (1.82,1.96) |
| 20 | 1.97 (1.89,2.04) | 2.09 (2.01,2.17) | 1.98 (1.92,2.06) |
| 21 | 2.04 (1.97,2.12) | 2.19 (2.12,2.27) | 2.08 (2.01,2.15) |
| 22 | 2.11 (2.04,2.19) | 2.29 (2.22,2.36) | 2.17 (2.10,2.24) |
| 23 | 2.19 (2.11,2.26) | 2.38 (2.31,2.46) | 2.26 (2.20,2.34) |
| 24 | 2.27 (2.19,2.35) | 2.48 (2.40,2.57) | 2.36 (2.29,2.44) |
| 25 | 2.37 (2.29,2.46) | 2.60 (2.51,2.69) | 2.47 (2.40,2.55) |
| 26 | 2.49 (2.41,2.58) | 2.72 (2.63,2.81) | 2.59 (2.52,2.67) |
| 27 | 2.63 (2.55,2.72) | 2.86 (2.77,2.94) | 2.72 (2.65,2.80) |
| 28 | 2.78 (2.70,2.87) | 3.00 (2.92,3.09) | 2.86 (2.78,2.94) |
| 29 | 2.94 (2.85,3.04) | 3.16 (3.06,3.26) | 3.00 (2.92,3.09) |
| 30 | 3.11 (3.01,3.21) | 3.32 (3.22,3.43) | 3.15 (3.06,3.25) |
| 31 | 3.27 (3.16,3.38) | 3.49 (3.38,3.60) | 3.31 (3.21,3.41) |
| 32 | 3.42 (3.31,3.53) | 3.66 (3.55,3.77) | 3.46 (3.36,3.57) |
| 33 | 3.58 (3.46,3.70) | 3.83 (3.71,3.95) | 3.62 (3.51,3.73) |
| 34 | 3.74 (3.61,3.88) | 4.01 (3.87,4.15) | 3.78 (3.66,3.90) |
| 35 | 3.93 (3.79,4.08) | 4.19 (4.05,4.34) | 3.93 (3.80,4.06) |
| 36 | 4.15 (4.00,4.30) | 4.38 (4.23,4.53) | 4.08 (3.95,4.21) |
| 37 | 4.36 (4.19,4.53) | 4.57 (4.40,4.75) | 4.23 (4.09,4.38) |
| 38 | 4.47 (4.29,4.66) | 4.75 (4.56,4.94) | 4.39 (4.22,4.55) |
| 39 | 4.39 (4.18,4.62) | 4.89 (4.68,5.11) | 4.55 (4.37,4.75) |
| 40 | 4.07 (3.67,4.52) | 4.99 (4.58,5.43) | 4.75 (4.41,5.11) |

© 2025 Clayton PK et al. *JAMA Network Open*.

**eTable 32.** Means of Fractional Thigh Volume by Gestational Week in Fetuses of Women with PHDI (T1), PHDI (T2), and PHDI (T3) among the Standard Population in the NICHD Fetal Growth Studies, 2009-2013.

| | Mean Fractional Thigh Volume, $cm^3$ (95% CI) | | |
|---|---|---|---|
| GA | T1 | T2 | T3 |
| 15 | 0.66 (0.60,0.74) | 0.65 (0.58,0.73) | 0.70 (0.64,0.76) |
| 16 | 1.11 (1.05,1.17) | 1.06 (1.00,1.13) | 1.10 (1.05,1.15) |
| 17 | 1.72 (1.66,1.78) | 1.64 (1.59,1.70) | 1.66 (1.61,1.71) |
| 18 | 2.51 (2.43,2.60) | 2.41 (2.34,2.49) | 2.39 (2.32,2.46) |
| 19 | 3.49 (3.37,3.60) | 3.39 (3.28,3.49) | 3.32 (3.22,3.42) |
| 20 | 4.63 (4.50,4.77) | 4.56 (4.44,4.68) | 4.45 (4.34,4.57) |
| 21 | 5.95 (5.79,6.11) | 5.92 (5.78,6.07) | 5.79 (5.65,5.93) |
| 22 | 7.44 (7.24,7.65) | 7.46 (7.28,7.65) | 7.33 (7.15,7.51) |
| 23 | 9.15 (8.90,9.40) | 9.17 (8.94,9.41) | 9.06 (8.84,9.28) |
| 24 | 11.07 (10.79,11.35) | 11.03 (10.77,11.30) | 10.98 (10.74,11.23) |
| 25 | 13.22 (12.91,13.53) | 13.07 (12.78,13.36) | 13.09 (12.82,13.35) |
| 26 | 15.59 (15.24,15.96) | 15.30 (14.96,15.64) | 15.40 (15.10,15.71) |
| 27 | 18.22 (17.78,18.66) | 17.78 (17.37,18.20) | 17.96 (17.59,18.34) |
| 28 | 21.11 (20.60,21.63) | 20.62 (20.13,21.11) | 20.84 (20.40,21.28) |
| 29 | 24.30 (23.76,24.85) | 23.93 (23.41,24.47) | 24.11 (23.64,24.60) |
| 30 | 27.83 (27.25,28.42) | 27.80 (27.22,28.39) | 27.84 (27.32,28.37) |
| 31 | 31.75 (31.06,32.47) | 32.23 (31.52,32.96) | 32.03 (31.39,32.67) |
| 32 | 36.15 (35.27,37.05) | 37.21 (36.30,38.14) | 36.66 (35.86,37.48) |
| 33 | 41.11 (40.09,42.15) | 42.68 (41.62,43.77) | 41.71 (40.77,42.67) |
| 34 | 46.69 (45.52,47.89) | 48.53 (47.35,49.73) | 47.13 (46.09,48.20) |
| 35 | 52.79 (51.30,54.32) | 54.57 (53.14,56.04) | 52.91 (51.69,54.17) |
| 36 | 59.24 (57.33,61.20) | 60.56 (58.75,62.43) | 59.05 (57.52,60.61) |
| 37 | 65.74 (63.53,68.03) | 66.19 (64.06,68.39) | 65.52 (63.69,67.40) |
| 38 | 71.92 (69.22,74.73) | 71.08 (68.70,73.54) | 72.32 (70.14,74.56) |
| 39 | 77.31 (72.54,82.40) | 74.83 (71.38,78.45) | 79.43 (76.24,82.76) |
| 40 | 81.38 (72.15,91.80) | 77.07 (70.70,84.03) | 86.86 (80.98,93.17) |

© 2025 Clayton PK et al. *JAMA Network Open*.

**eTable 33.** Means of Fractional Lean Thigh Volume by Gestational Week in Fetuses of Women with PHDI (T1), PHDI (T2), and PHDI (T3) among the Standard Population in the NICHD Fetal Growth Studies, 2009-2013.

| | Mean Fractional Lean Thigh Volume, $cm^3$ (95% CI) | | |
|---|---|---|---|
| GA | T1 | T2 | T3 |
| 15 | 0.33 (0.28,0.38) | 0.33 (0.29,0.38) | 0.35 (0.32,0.39) |
| 16 | 0.56 (0.52,0.60) | 0.56 (0.52,0.61) | 0.57 (0.54,0.61) |
| 17 | 0.89 (0.85,0.93) | 0.90 (0.86,0.94) | 0.89 (0.85,0.92) |
| 18 | 1.34 (1.28,1.39) | 1.35 (1.30,1.40) | 1.31 (1.26,1.35) |
| 19 | 1.90 (1.82,1.98) | 1.92 (1.85,1.99) | 1.85 (1.79,1.92) |
| 20 | 2.57 (2.47,2.67) | 2.62 (2.53,2.70) | 2.52 (2.44,2.60) |
| 21 | 3.35 (3.24,3.46) | 3.42 (3.33,3.52) | 3.32 (3.22,3.41) |
| 22 | 4.23 (4.10,4.37) | 4.33 (4.21,4.45) | 4.23 (4.11,4.35) |
| 23 | 5.21 (5.04,5.38) | 5.33 (5.17,5.49) | 5.25 (5.10,5.40) |
| 24 | 6.31 (6.11,6.51) | 6.42 (6.23,6.61) | 6.36 (6.19,6.54) |
| 25 | 7.52 (7.30,7.74) | 7.59 (7.38,7.80) | 7.55 (7.37,7.75) |
| 26 | 8.84 (8.60,9.08) | 8.85 (8.62,9.08) | 8.83 (8.63,9.04) |
| 27 | 10.27 (9.99,10.55) | 10.22 (9.95,10.49) | 10.21 (9.97,10.45) |
| 28 | 11.80 (11.46,12.14) | 11.71 (11.39,12.03) | 11.70 (11.42,11.99) |
| 29 | 13.42 (13.06,13.80) | 13.38 (13.02,13.74) | 13.36 (13.04,13.68) |
| 30 | 15.16 (14.79,15.54) | 15.26 (14.89,15.64) | 15.22 (14.88,15.56) |
| 31 | 17.04 (16.62,17.47) | 17.35 (16.92,17.78) | 17.28 (16.90,17.68) |
| 32 | 19.13 (18.60,19.67) | 19.63 (19.09,20.18) | 19.55 (19.06,20.04) |
| 33 | 21.49 (20.87,22.14) | 22.07 (21.42,22.73) | 21.98 (21.41,22.57) |
| 34 | 24.23 (23.54,24.93) | 24.6 (23.91,25.31) | 24.56 (23.94,25.19) |
| 35 | 27.27 (26.44,28.13) | 27.17 (26.39,27.98) | 27.24 (26.55,27.96) |
| 36 | 30.48 (29.40,31.59) | 29.70 (28.73,30.71) | 30.00 (29.15,30.88) |
| 37 | 33.62 (32.36,34.94) | 32.10 (30.96,33.28) | 32.81 (31.79,33.86) |
| 38 | 36.41 (34.94,37.95) | 34.26 (33.02,35.56) | 35.61 (34.43,36.84) |
| 39 | 38.49 (35.98,41.17) | 36.08 (34.32,37.93) | 38.37 (36.66,40.16) |
| 40 | 39.47 (34.59,45.05) | 37.45 (34.06,41.17) | 41.03 (37.82,44.51) |

© 2025 Clayton PK et al. *JAMA Network Open*.

**eTable 34.** Means of Fractional Fat Thigh Volume by Gestational Week in Fetuses of Women with PHDI (T1), PHDI (T2), and PHDI (T3) among the Standard Population in the NICHD Fetal Growth Studies, 2009-2013.

| | Mean Fractional Fat Thigh Volume, $cm^3$ (95% CI) | | |
|---|---|---|---|
| GA | T1 | T2 | T3 |
| 15 | 0.30 (0.26,0.35) | 0.32 (0.28,0.37) | 0.32 (0.29,0.36) |
| 16 | 0.52 (0.47,0.56) | 0.51 (0.47,0.55) | 0.51 (0.48,0.54) |
| 17 | 0.81 (0.77,0.85) | 0.76 (0.73,0.80) | 0.76 (0.73,0.79) |
| 18 | 1.18 (1.13,1.24) | 1.09 (1.04,1.13) | 1.08 (1.04,1.12) |
| 19 | 1.62 (1.55,1.69) | 1.48 (1.42,1.54) | 1.47 (1.42,1.53) |
| 20 | 2.10 (2.01,2.19) | 1.94 (1.88,2.01) | 1.93 (1.86,2.00) |
| 21 | 2.62 (2.53,2.72) | 2.48 (2.40,2.55) | 2.46 (2.38,2.54) |
| 22 | 3.19 (3.08,3.31) | 3.08 (2.99,3.18) | 3.06 (2.97,3.15) |
| 23 | 3.83 (3.70,3.97) | 3.76 (3.64,3.89) | 3.74 (3.63,3.86) |
| 24 | 4.59 (4.43,4.75) | 4.53 (4.38,4.68) | 4.53 (4.39,4.66) |
| 25 | 5.49 (5.31,5.66) | 5.40 (5.24,5.57) | 5.42 (5.27,5.57) |
| 26 | 6.54 (6.34,6.74) | 6.40 (6.21,6.59) | 6.45 (6.28,6.62) |
| 27 | 7.76 (7.52,8.00) | 7.55 (7.32,7.78) | 7.63 (7.43,7.84) |
| 28 | 9.16 (8.86,9.46) | 8.89 (8.61,9.17) | 8.99 (8.74,9.25) |
| 29 | 10.74 (10.41,11.08) | 10.47 (10.16,10.80) | 10.57 (10.28,10.86) |
| 30 | 12.52 (12.17,12.88) | 12.37 (12.02,12.72) | 12.40 (12.09,12.72) |
| 31 | 14.52 (14.10,14.95) | 14.60 (14.17,15.03) | 14.50 (14.12,14.88) |
| 32 | 16.77 (16.23,17.33) | 17.18 (16.62,17.75) | 16.86 (16.37,17.36) |
| 33 | 19.32 (18.66,20.00) | 20.11 (19.42,20.83) | 19.46 (18.86,20.08) |
| 34 | 22.21 (21.45,22.98) | 23.37 (22.60,24.17) | 22.27 (21.60,22.96) |
| 35 | 25.38 (24.44,26.36) | 26.82 (25.90,27.79) | 25.28 (24.51,26.08) |
| 36 | 28.74 (27.51,30.03) | 30.28 (29.08,31.54) | 28.52 (27.55,29.53) |
| 37 | 32.09 (30.64,33.61) | 33.47 (32.03,34.98) | 32.00 (30.81,33.24) |
| 38 | 35.20 (33.45,37.04) | 36.05 (34.45,37.72) | 35.77 (34.34,37.25) |
| 39 | 37.77 (34.65,41.16) | 37.66 (35.35,40.11) | 39.86 (37.74,42.09) |
| 40 | 39.47 (33.35,46.71) | 37.98 (33.68,42.83) | 44.35 (40.29,48.81) |

© 2025 Clayton PK et al. *JAMA Network Open*.

**eTable 35.** Means of Maximum Thigh Subcutaneous Tissue Thickness (SCTT) by Gestational Week in Fetuses of Women with PHDI (T1), PHDI (T2), and PHDI (T3) among the Standard Population in the NICHD Fetal Growth Studies, 2009-2013.

| | Mean Maximum Thigh SCTT, cm (95% CI) | | |
|---|---|---|---|
| GA | T1 | T2 | T3 |
| 15 | 0.15 (0.13,0.17) | 0.17 (0.15,0.20) | 0.16 (0.15,0.18) |
| 16 | 0.18 (0.17,0.20) | 0.19 (0.17,0.20) | 0.18 (0.17,0.20) |
| 17 | 0.21 (0.20,0.22) | 0.21 (0.20,0.22) | 0.20 (0.20,0.22) |
| 18 | 0.24 (0.23,0.25) | 0.23 (0.22,0.24) | 0.23 (0.22,0.24) |
| 19 | 0.26 (0.25,0.28) | 0.25 (0.24,0.26) | 0.25 (0.24,0.26) |
| 20 | 0.28 (0.27,0.30) | 0.27 (0.26,0.28) | 0.28 (0.27,0.29) |
| 21 | 0.30 (0.29,0.32) | 0.30 (0.29,0.31) | 0.30 (0.29,0.31) |
| 22 | 0.32 (0.31,0.34) | 0.33 (0.31,0.34) | 0.32 (0.31,0.34) |
| 23 | 0.34 (0.33,0.36) | 0.35 (0.34,0.37) | 0.35 (0.33,0.36) |
| 24 | 0.36 (0.35,0.38) | 0.38 (0.37,0.39) | 0.37 (0.36,0.38) |
| 25 | 0.39 (0.37,0.40) | 0.41 (0.39,0.42) | 0.39 (0.38,0.41) |
| 26 | 0.41 (0.40,0.43) | 0.43 (0.42,0.45) | 0.42 (0.40,0.43) |
| 27 | 0.44 (0.43,0.46) | 0.46 (0.44,0.48) | 0.44 (0.43,0.45) |
| 28 | 0.48 (0.46,0.50) | 0.49 (0.47,0.51) | 0.47 (0.45,0.48) |
| 29 | 0.51 (0.49,0.53) | 0.52 (0.50,0.54) | 0.50 (0.48,0.51) |
| 30 | 0.55 (0.53,0.57) | 0.56 (0.54,0.58) | 0.53 (0.52,0.55) |
| 31 | 0.59 (0.57,0.61) | 0.59 (0.57,0.62) | 0.57 (0.56,0.59) |
| 32 | 0.62 (0.60,0.65) | 0.63 (0.61,0.66) | 0.62 (0.60,0.64) |
| 33 | 0.66 (0.63,0.69) | 0.68 (0.65,0.70) | 0.66 (0.64,0.69) |
| 34 | 0.70 (0.67,0.72) | 0.72 (0.69,0.75) | 0.71 (0.69,0.74) |
| 35 | 0.73 (0.70,0.76) | 0.76 (0.73,0.79) | 0.76 (0.73,0.79) |
| 36 | 0.77 (0.73,0.81) | 0.80 (0.76,0.84) | 0.81 (0.78,0.84) |
| 37 | 0.81 (0.77,0.86) | 0.83 (0.79,0.88) | 0.85 (0.81,0.89) |
| 38 | 0.87 (0.82,0.92) | 0.86 (0.82,0.91) | 0.89 (0.84,0.93) |
| 39 | 0.93 (0.85,1.02) | 0.88 (0.82,0.94) | 0.91 (0.85,0.97) |
| 40 | 1.02 (0.86,1.22) | 0.89 (0.79,1.01) | 0.91 (0.82,1.02) |

© 2025 Clayton PK et al. *JAMA Network Open*.

**eTable 36.** Means of Cerebellar Volume by Gestational Week in Fetuses of Women with PHDI (T1), PHDI (T2), and PHDI (T3) among the Standard Population in the NICHD Fetal Growth Studies, 2009-2013.

| | Mean Cerebellar Volume, $cm^3$ (95% CI) | | |
|---|---|---|---|
| GA | T1 | T2 | T3 |
| 15 | 0.43 (0.35,0.51) | 0.47 (0.39,0.56) | 0.39 (0.33,0.45) |
| 16 | 0.57 (0.52,0.62) | 0.62 (0.57,0.68) | 0.54 (0.50,0.58) |
| 17 | 0.74 (0.70,0.78) | 0.80 (0.76,0.84) | 0.72 (0.69,0.76) |
| 18 | 0.96 (0.91,1.01) | 1.00 (0.95,1.06) | 0.94 (0.89,0.99) |
| 19 | 1.22 (1.16,1.28) | 1.24 (1.18,1.31) | 1.20 (1.14,1.26) |
| 20 | 1.53 (1.47,1.61) | 1.52 (1.45,1.59) | 1.49 (1.42,1.56) |
| 21 | 1.91 (1.82,1.99) | 1.84 (1.77,1.92) | 1.83 (1.76,1.91) |
| 22 | 2.34 (2.23,2.45) | 2.23 (2.14,2.33) | 2.23 (2.14,2.32) |
| 23 | 2.84 (2.71,2.97) | 2.70 (2.58,2.81) | 2.71 (2.60,2.82) |
| 24 | 3.41 (3.27,3.55) | 3.26 (3.14,3.39) | 3.28 (3.16,3.40) |
| 25 | 4.06 (3.90,4.22) | 3.94 (3.80,4.09) | 3.94 (3.81,4.08) |
| 26 | 4.80 (4.61,4.99) | 4.74 (4.56,4.91) | 4.71 (4.56,4.88) |
| 27 | 5.63 (5.41,5.87) | 5.66 (5.45,5.89) | 5.59 (5.39,5.80) |
| 28 | 6.58 (6.32,6.84) | 6.72 (6.47,6.98) | 6.56 (6.33,6.80) |
| 29 | 7.65 (7.38,7.92) | 7.90 (7.63,8.18) | 7.63 (7.38,7.88) |
| 30 | 8.82 (8.51,9.14) | 9.19 (8.88,9.52) | 8.77 (8.49,9.06) |
| 31 | 10.07 (9.69,10.47) | 10.56 (10.18,10.96) | 9.98 (9.65,10.33) |
| 32 | 11.36 (10.93,11.81) | 11.95 (11.51,12.41) | 11.25 (10.87,11.65) |
| 33 | 12.67 (12.18,13.17) | 13.33 (12.84,13.83) | 12.57 (12.15,13.01) |
| 34 | 14.00 (13.39,14.63) | 14.66 (14.09,15.25) | 13.95 (13.46,14.46) |
| 35 | 15.36 (14.59,16.17) | 15.91 (15.22,16.64) | 15.37 (14.77,16.00) |
| 36 | 16.78 (15.86,17.76) | 17.07 (16.22,17.95) | 16.86 (16.12,17.62) |
| 37 | 18.30 (17.18,19.50) | 18.10 (17.07,19.18) | 18.41 (17.53,19.33) |
| 38 | 19.97 (18.22,21.90) | 18.99 (17.59,20.49) | 20.05 (18.89,21.27) |
| 39 | 21.86 (18.62,25.66) | 19.73 (17.53,22.20) | 21.79 (19.97,23.79) |
| 40 | 24.06 (18.30,31.62) | 20.32 (16.79,24.60) | 23.68 (20.55,27.28) |

© 2025 Clayton PK et al. *JAMA Network Open*.

**eTable 37.** Means of Average Lung Volume by Gestational Week in Fetuses of Women with PHDI (T1), PHDI (T2), and PHDI (T3) among the Standard Population in the NICHD Fetal Growth Studies, 2009-2013.

| | Mean Lung Volume, $mm^2$ (95% CI) | | |
|---|---|---|---|
| GA | T1 | T2 | T3 |
| 15 | 1.62 (0.79,3.34) | 1.36 (0.69,2.69) | 1.08 (0.69,1.68) |
| 16 | 1.82 (1.41,2.35) | 1.91 (1.58,2.32) | 1.55 (1.30,1.85) |
| 17 | 2.22 (1.82,2.71) | 2.52 (1.99,3.18) | 2.12 (1.80,2.49) |
| 18 | 2.86 (2.42,3.38) | 3.18 (2.55,3.95) | 2.77 (2.39,3.22) |
| 19 | 3.72 (3.14,4.41) | 3.92 (3.29,4.69) | 3.51 (3.00,4.11) |
| 20 | 4.74 (3.85,5.84) | 4.84 (4.01,5.85) | 4.37 (3.65,5.23) |
| 21 | 5.86 (4.77,7.20) | 5.99 (4.98,7.20) | 5.38 (4.52,6.40) |
| 22 | 7.06 (5.94,8.40) | 7.38 (6.31,8.62) | 6.56 (5.65,7.60) |
| 23 | 8.35 (7.21,9.66) | 9.00 (7.90,10.25) | 7.93 (7.01,8.98) |
| 24 | 9.74 (8.43,11.25) | 10.81 (9.52,12.27) | 9.55 (8.47,10.76) |
| 25 | 11.27 (9.72,13.08) | 12.71 (11.13,14.5) | 11.44 (10.11,12.94) |
| 26 | 12.97 (11.22,14.98) | 14.65 (12.83,16.72) | 13.62 (12.03,15.42) |
| 27 | 14.83 (12.92,17.03) | 16.61 (14.57,18.94) | 16.08 (14.21,18.19) |
| 28 | 16.88 (14.70,19.39) | 18.61 (16.29,21.26) | 18.75 (16.53,21.27) |
| 29 | 19.12 (16.55,22.09) | 20.67 (18.01,23.73) | 21.54 (18.89,24.57) |
| 30 | 21.58 (18.62,25.00) | 22.83 (19.85,26.26) | 24.32 (21.25,27.83) |
| 31 | 24.24 (20.90,28.12) | 25.15 (21.92,28.87) | 26.93 (23.54,30.81) |
| 32 | 27.07 (23,31.85.00) | 27.59 (23.92,31.83) | 29.38 (25.64,33.68) |
| 33 | 29.93 (24.67,36.30) | 30.05 (25.58,35.30) | 31.76 (27.48,36.71) |
| 34 | 32.67 (26.11,40.88) | 32.41 (26.95,38.97) | 34.21 (29.25,40.02) |
| 35 | 35.11 (27.65,44.59) | 34.51 (28.25,42.17) | 36.94 (31.29,43.60) |
| 36 | 37.04 (29.38,46.69) | 36.20 (29.62,44.24) | 40.19 (33.94,47.59) |
| 37 | 38.22 (30.33,48.17) | 37.29 (30.77,45.20) | 44.33 (37.13,52.94) |
| 38 | 38.48 (27.76,53.33) | 37.63 (30.15,46.96) | 49.86 (39.82,62.43) |
| 39 | 37.67 (21.32,66.56) | 37.08 (26.10,52.69) | 57.49 (40.76,81.09) |
| 40 | 35.76 (13.78,92.75) | 35.61 (19.72,64.30) | 68.36 (39.73,117.64) |

© 2025 Clayton PK et al. *JAMA Network Open*.

**eTable 38.** Means of Kidney by Gestational Week in Fetuses of Women with PHDI (T1), PHDI (T2), and PHDI (T3) among the Standard Population in the NICHD Fetal Growth Studies, 2009-2013.

| | Mean Kidney Volume, $mm^2$ (95% CI) | | |
|---|---|---|---|
| GA | T1 | T2 | T3 |
| 15 | 0.20 (0.11,0.34) | 0.34 (0.21,0.53) | 0.41 (0.26,0.64) |
| 16 | 0.37 (0.29,0.47) | 0.49 (0.39,0.60) | 0.47 (0.39,0.57) |
| 17 | 0.59 (0.50,0.69) | 0.67 (0.58,0.78) | 0.57 (0.50,0.64) |
| 18 | 0.81 (0.70,0.94) | 0.90 (0.79,1.03) | 0.71 (0.62,0.82) |
| 19 | 1.01 (0.89,1.16) | 1.17 (1.05,1.32) | 0.92 (0.81,1.04) |
| 20 | 1.21 (1.07,1.36) | 1.49 (1.34,1.66) | 1.18 (1.05,1.32) |
| 21 | 1.43 (1.26,1.63) | 1.85 (1.64,2.09) | 1.50 (1.33,1.70) |
| 22 | 1.73 (1.52,1.96) | 2.26 (2.00,2.56) | 1.87 (1.65,2.12) |
| 23 | 2.11 (1.87,2.38) | 2.71 (2.42,3.04) | 2.28 (2.04,2.55) |
| 24 | 2.58 (2.30,2.90) | 3.20 (2.89,3.55) | 2.74 (2.49,3.02) |
| 25 | 3.15 (2.78,3.57) | 3.73 (3.37,4.13) | 3.25 (2.97,3.56) |
| 26 | 3.79 (3.29,4.36) | 4.28 (3.83,4.79) | 3.81 (3.47,4.19) |
| 27 | 4.46 (3.86,5.15) | 4.85 (4.31,5.45) | 4.44 (4.03,4.89) |
| 28 | 5.12 (4.48,5.85) | 5.42 (4.85,6.05) | 5.14 (4.70,5.62) |
| 29 | 5.77 (5.12,6.49) | 6.00 (5.42,6.64) | 5.90 (5.42,6.42) |
| 30 | 6.39 (5.69,7.18) | 6.57 (5.95,7.26) | 6.71 (6.11,7.36) |
| 31 | 7.01 (6.19,7.94) | 7.14 (6.43,7.93) | 7.54 (6.77,8.39) |
| 32 | 7.65 (6.72,8.70) | 7.71 (6.92,8.59) | 8.36 (7.45,9.38) |
| 33 | 8.34 (7.39,9.41) | 8.28 (7.44,9.20) | 9.13 (8.17,10.20) |
| 34 | 9.11 (8.12,10.23) | 8.86 (7.94,9.90) | 9.84 (8.85,10.95) |
| 35 | 9.98 (8.81,11.32) | 9.50 (8.38,10.77) | 10.50 (9.38,11.76) |
| 36 | 10.98 (9.56,12.62) | 10.21 (8.89,11.72) | 11.13 (9.81,12.61) |
| 37 | 12.15 (10.52,14.02) | 11.04 (9.62,12.66) | 11.74 (10.30,13.39) |
| 38 | 13.52 (11.42,16.00) | 12.04 (10.50,13.81) | 12.38 (10.85,14.14) |
| 39 | 15.16 (11.34,20.25) | 13.30 (10.77,16.44) | 13.09 (10.97,15.62) |
| 40 | 17.14 (10.00,29.37) | 14.92 (9.95,22.37) | 13.91 (10.15,19.07) |

© 2025 Clayton PK et al. *JAMA Network Open*.

**eTable 39.** Means of Liver by Gestational Week in Fetuses of Women with PHDI (T1), PHDI (T2), and PHDI (T3) among the Standard Population in the NICHD Fetal Growth Studies, 2009-2013.

| | Mean Liver Volume, $mm^2$ (95% CI) | | |
|---|---|---|---|
| GA | T1 | T2 | T3 |
| 15 | 3.38 (2.69,4.26) | 2.82 (2.22,3.58) | 2.89 (2.40,3.48) |
| 16 | 4.19 (3.71,4.73) | 3.96 (3.49,4.49) | 3.96 (3.58,4.38) |
| 17 | 5.28 (4.88,5.71) | 5.39 (4.99,5.83) | 5.33 (4.96,5.73) |
| 18 | 6.73 (6.23,7.27) | 7.16 (6.66,7.69) | 7.05 (6.55,7.59) |
| 19 | 8.64 (8.00,9.33) | 9.27 (8.65,9.95) | 9.17 (8.54,9.86) |
| 20 | 11.09 (10.34,11.89) | 11.76 (11.05,12.52) | 11.74 (11.00,12.53) |
| 21 | 14.14 (13.27,15.06) | 14.64 (13.84,15.50) | 14.79 (13.95,15.67) |
| 22 | 17.79 (16.72,18.94) | 17.94 (16.93,19.01) | 18.32 (17.32,19.39) |
| 23 | 21.98 (20.63,23.42) | 21.69 (20.41,23.04) | 22.35 (21.12,23.65) |
| 24 | 26.60 (25.03,28.26) | 25.91 (24.43,27.47) | 26.85 (25.45,28.34) |
| 25 | 31.59 (29.87,33.41) | 30.63 (29.02,32.34) | 31.79 (30.25,33.42) |
| 26 | 36.90 (34.99,38.91) | 35.89 (34.08,37.79) | 37.13 (35.42,38.93) |
| 27 | 42.45 (40.22,44.79) | 41.71 (39.56,43.97) | 42.81 (40.80,44.92) |
| 28 | 48.19 (45.60,50.93) | 48.14 (45.57,50.86) | 48.76 (46.40,51.23) |
| 29 | 54.09 (51.28,57.05) | 55.25 (52.37,58.30) | 54.90 (52.31,57.63) |
| 30 | 60.12 (57.28,63.10) | 63.03 (59.99,66.23) | 61.20 (58.52,64.01) |
| 31 | 66.27 (63.23,69.45) | 71.23 (67.87,74.76) | 67.69 (64.80,70.72) |
| 32 | 72.54 (68.96,76.30) | 79.47 (75.46,83.69) | 74.47 (71.10,78.00) |
| 33 | 78.97 (74.85,83.31) | 87.20 (82.58,92.07) | 81.66 (77.79,85.73) |
| 34 | 85.62 (81.29,90.17) | 93.84 (89.11,98.82) | 89.48 (85.35,93.80) |
| 35 | 92.60 (87.87,97.59) | 99.49 (94.64,104.59) | 97.98 (93.52,102.64) |
| 36 | 100.13 (94.31,106.30) | 105.05 (99.43,110.99) | 107.10 (101.84,112.63) |
| 37 | 108.44 (101.52,115.84) | 111.66 (105.13,118.59) | 116.72 (110.59,123.18) |
| 38 | 117.87 (109.87,126.45) | 120.77 (113.60,128.38) | 126.66 (119.81,133.92) |
| 39 | 128.85 (116.18,142.91) | 134.36 (123.99,145.60) | 136.71 (126.63,147.60) |
| 40 | 141.94 (116.12,173.48) | 155.44 (132.99,181.68) | 146.58 (126.70,169.57) |

© 2025 Clayton PK et al. *JAMA Network Open*.

**eTable 40.** Global and Weekly Comparisons of Longitudinal, Two-dimensional and Three-dimensional Fetal Growth, Body Composition, and Organ Volumes across PHDI tertiles among the Standard Population; NICHD Fetal Growth Studies (n=1034) †

| | Global Comparison | | Weekly Comparisons | | | |
|---|---|---|---|---|---|---|
| | | | Tertile 2 vs. Tertile 1 | | Tertile 3 vs. Tertile 1 | |
| **Outcome** | n | p-value | GA | Direction of Association | GA | Direction of Association |
| **Body Composition** | | | | | | |
| Estimated fetal weight, g | 1022 | 0.08 | 33-38 | Larger | 35 | Larger |
| **2D Measures** | | | | | | |
| Head circumference, mm | 1025 | 0.10 | - | - | - | - |
| Abdominal circumference, mm | 1025 | 0.61 | 34-36 | Larger | - | - |
| Average Femur length, mm | 1026 | 0.19 | - | - | - | - |
| Average Humerus length, mm | 1024 | 0.21 | - | - | - | - |
| **3D Measures** | | | | | | |
| Fractional arm volume, $cm^3$ | 675 | 0.66 | - | - | - | - |
| Fractional lean arm volume, $cm^3$ | 576 | 0.78 | - | - | - | - |
| Fractional fat arm volume, $cm^3$ | 576 | 0.22 | 35-37 | Larger | - | - |
| Maximum arm SCTT, cm | 621 | 0.74 | - | - | - | - |
| Abdominal area, $mm^2$ | 957 | 0.29 | - | - | - | - |
| Maximum abdominal SCTT, cm | 913 | 0.07 | 21-33, 39-40 | Larger, Larger | - | - |
| Fractional thigh volume, $cm^3$ | 674 | 0.41 | - | - | - | - |
| Fractional lean thigh volume, $cm^3$ | 594 | 0.85 | - | - | - | - |
| Fractional fat thigh volume, $cm^3$ | 593 | 0.23 | - | - | - | - |
| Maximum thigh SCTT, cm | 621 | 0.62 | - | - | - | - |
| **Organ Volumes** | | | | | | |
| Cerebellar volume, $cm^3$ | 566 | 0.21 | - | - | - | - |
| Average lung volume, $cm^3$ | 94 | 0.67 | - | - | - | - |
| Average kidney volume, $cm^3$ | 225 | 0.72 | - | - | - | - |
| Liver volume, $cm^3$ | 479 | 0.65 | - | - | - | - |

GA = gestational age; SCTT = subcutaneous tissue thickness.
Linear mixed models were adjusted for: maternal age, race/ethnicity, parity, pre-pregnancy BMI, education, infant sex, job status, and married.
Dashes indicate no gestational age for which pairwise comparisons were different.
Reported p-values are corrected for multiple comparisons using Tukey's Method.
†Women without an obese pre-pregnancy BMI and without a pregnancy complication in the current pregnancy.

© 2025 Clayton PK et al. *JAMA Network Open*.

**eTable 41.** Global and Weekly Comparisons of Longitudinal, Two-dimensional and Three-dimensional Fetal Growth, Body Composition, and Organ Volumes across PHDI tertiles correcting for multiple comparisons; NICHD Fetal Growth Studies-Singletons (n=1464)

| | Global Comparison | | Weekly Comparisons | | | |
|---|---|---|---|---|---|---|
| | | | Tertile 2 vs. Tertile 1 | | Tertile 3 vs. Tertile 1 | |
| **Outcome** | n | p-value | GA | Direction of Association | GA | Direction of Association |
| **Body Composition** | | | | | | |
| Estimated fetal weight, g | 1443 | 0.01 | 33-39 | Larger | 32-36, 38-40 | Larger, Larger |
| **2D Measures** | | | | | | |
| Head circumference, mm | 1449 | 0.04 | - | - | 38 | Larger |
| Abdominal circumference, mm | 1448 | 0.30 | - | - | 32-35 | Larger |
| Average Femur length, mm | 1449 | 0.04 | 15-16 | Larger | - | - |
| Average Humerus length, mm | 1450 | 0.02 | - | - | - | - |
| **3D Measures** | | | | | | |
| Fractional arm volume, $cm^3$ | 915 | 0.74 | - | - | - | - |
| Fractional lean arm volume, $cm^3$ | 775 | 0.21 | 34-38 | Smaller | 34-36 | Smaller |
| Fractional fat arm volume, $cm^3$ | 775 | 0.35 | 35-37 | Larger | 37 | Larger |
| Maximum arm SCTT, cm | 846 | 0.61 | - | - | - | - |
| Abdominal area, $mm^2$ | 1360 | 0.16 | - | - | - | - |
| Maximum abdominal SCTT, cm | 1290 | 0.43 | 27-29, 38 | Larger, Larger | - | - |
| Fractional thigh volume, $cm^3$ | 910 | 0.56 | - | - | - | - |
| Fractional lean thigh volume, $cm^3$ | 801 | 0.39 | 37-38 | Smaller | - | - |
| Fractional fat thigh volume, $cm^3$ | 800 | 0.64 | - | - | - | - |
| Maximum thigh SCTT, cm | 844 | 0.65 | - | - | - | - |
| **Organ Volumes** | | | | | | |
| Cerebellar volume, $cm^3$ | 772 | 0.64 | - | - | - | - |
| Average lung volume, $cm^3$ | 128 | 0.50 | 27 | Larger | - | - |
| Average kidney volume, $cm^3$ | 540 | 0.35 | 15-16, 23-25 | Larger, Larger | - | - |
| Liver volume, $cm^3$ | 652 | 0.74 | - | - | - | - |

GA = gestational age; SCTT = subcutaneous tissue thickness.
Linear mixed models were adjusted for: maternal age, race/ethnicity, parity, pre-pregnancy BMI, education, infant sex, job status, and married.
Dashes indicate no gestational age for which pairwise comparisons were different.
Reported p-values are corrected for multiple comparisons using Tukey's Method.

© 2025 Clayton PK et al. *JAMA Network Open*.
