# Supplementary material for: Adherence to the Planetary Health Diet Index and Fetal Body Composition
Source: JAMA Netw Open. 2025 Dec 17;8(12):e2544153. doi: 10.1001/jamanetworkopen.2025.44153 (PMC12712731; doi:10.1001/jamanetworkopen.2025.44153)
Supplement: Supplement 2. — Data Sharing Statement [file jamanetwopen-e2544153-s002.pdf]

Data Sharing Statement

Clayton. Adherence to the Planetary Health Diet Index and Fetal Body Composition. *JAMA Netw Open*. Published November 18, 2025. doi:10.1001/jamanetworkopen.2025.44153

Data
Data available: Yes
Data types: Deidentified participant data, Data dictionary
How to access data: The data, along with a set of guidelines for researchers applying for the data, will be posted to a data-sharing site, the NICHD/DIPHR Biospecimen Repository Access and Data Sharing [https://brads.nichd.nih.gov] (BRADS).
When available: With publication

Supporting Documents
Document types: None

Additional Information
Who can access the data: anyone requesting the data, researchers whose proposed use of the data has been approved through a data use agreement (DUA).
Types of analyses: For any purpose or for a specified purpose
Mechanisms of data availability: With investigator support, after approval of a proposal, and with a signed data access agreement
